# Fabrication of α-Fe_2_O_3_ Nanoparticles/g-C_3_N_4_ Direct Z-Scheme Heterojunction of Durable Photocatalytic Activity

**DOI:** 10.1021/acsanm.5c00991

**Published:** 2025-04-29

**Authors:** Alejandro Galán-González, Isaías Fernández, Nestor J. Zaluzec, Sofie Cambré, Raul Arenal, Ana M. Benito, Wolfgang K. Maser

**Affiliations:** † Instituto de Carboquímica (ICB-CSIC), C/Miguel Luesma Castán 4, 50018Zaragoza, Spain; ‡ Pritzker School of Molecular Engineering and Argonne National Laboratory/Photon Science Directorate, University of Chicago, Lemont 60637, Illinois, United States; § Theory and Spectroscopy of Molecules and Materials, Department of Physics, University of Antwerp, 2610Antwerp, Belgium; ∥ Instituto de Nanociencia y Materiales de Aragón (INMA), CSIC-Universidad de Zaragoza, C/Pedro Cerbuna 12, 50009Zaragoza, Spain; ⊥ Laboratorio de Microscopias Avanzadas (LMA), Universidad de Zaragoza, C/Mariano Esquillor s/n, 50018Zaragoza, Spain; # ARAID Foundation, 50018Zaragoza, Spain

**Keywords:** nanohybrids, hematite nanoparticles, graphitic carbon nitride, Z-scheme heterojunction, photocatalysts

## Abstract

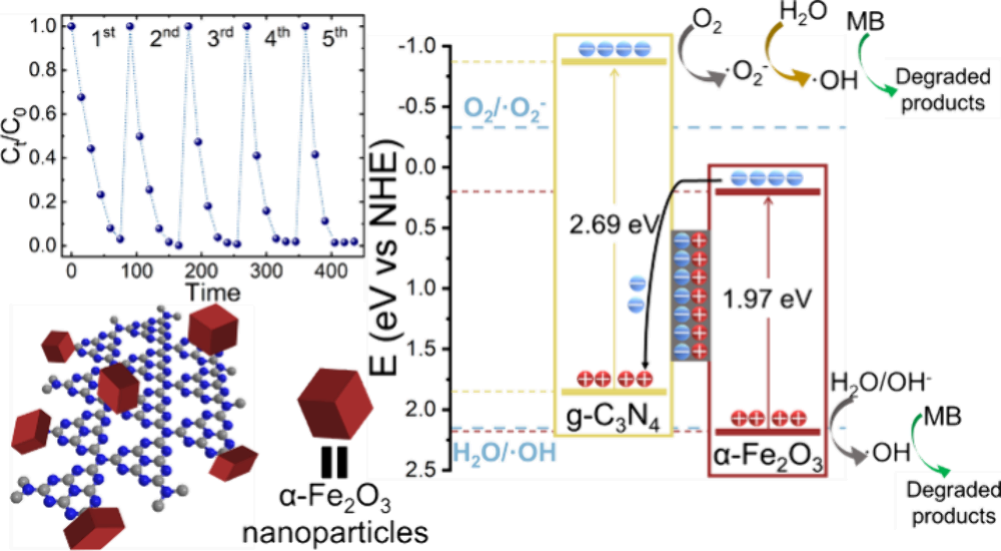

The fabrication of a nanohybrid photocatalyst that combines α-Fe_2_O_3_ nanoparticles with graphitic carbon nitride (g-C_3_N_4_) is reported. The ensuing direct Z-scheme heterojunction greatly boosts the photocatalytic activity of the α-Fe_2_O_3_/g-C_3_N_4_ nanohybrids. This results in organic dye degradation rates more than two times higher than its individual components, promoted by the efficient charge separation and transfer of the Z-scheme heterojunction mechanism of the nanohybrid photocatalyst. In addition, recyclability tests show an outstanding stability of the nanohybrids spanning five consecutive dye degradation experiments, during which the degradation rate is slightly improved. The origin of the improved photocatalytic performance of the nanohybrid lies in the intimate interaction between α-Fe_2_O_3_ and g-C_3_N_4_ afforded by the two-step fabrication process, which enables the direct and controlled growth of α-Fe_2_O_3_ nanoparticles on g-C_3_N_4_. A first ultrasound impregnation step promotes the effective anchoring of stable Fe species via Fe−N and C−N/C−O bonding, while a second microwave phase conversion step induces the subsequent growth of α-Fe_2_O_3_ nanoparticles on the g-C_3_N_4_ sheets. Careful control of the FeCl_3_ precursor concentration up to a threshold value of 0.25 M during impregnation enables complete control over their size and phase. This approach clearly highlights the benefits of microwave reactor systems in the fabrication of hematite-based Z-scheme photocatalytic, overcoming the limitations of conventional thermal treatment technology.

## 1 Introduction

Hematite (α-Fe_2_O_3_) is a metal oxide that holds significant promise for the photocatalytic removal of pollutants from water due to its ability to act as a Fenton’s reagent^1−3^ under UV light and in the presence of H_2_O_2_ as co-oxidant, facilitating the degradation of contaminants like dyes, pesticides or pharmaceuticals, while minimizing the emission of toxic byproducts.^4−7^ However, α-Fe_2_O_3_ still suffers from shortcomings that hinder its applicability in photocatalytic systems, such as an extremely short lifetime of photogenerated charge carriers, short hole diffusion lengths of a few nanometers, and a tendency to form large, poorly dispersed nanoparticles, limiting the number of available active surface sites.^8−12^ To address these drawbacks, various strategies like facet engineering,^13,14^ doping,^15,16^ and heterostructuring^17−19^ have been explored.

Among these approaches, the formation of α-Fe_2_O_3_-based heterojunction photocatalysts through the combination of hematite with other semiconductors offers significant potential to overcome the aforementioned limitations and boost overall photocatalytic activity.^20^ In this sense, direct Z-scheme photocatalytic systems have become the most promising type of heterojunction since they offer the best efficacy in the separation and transfer of the photogenerated charge carriers due to the Z-shaped charge-carrier migration pathway within both semiconductors.^21−23^ The interest in this charge transfer mechanism has led to the study of a variety of hematite-based direct Z-scheme photocatalytic systems by coupling α-Fe_2_O_3_ with perovskites,^24^ other metal oxides,^25,26^ semiconductors,^27,28^ or graphitic carbon nitride (g-C_3_N_4_).^29,30^

Among these, g-C_3_N_4_ has received significant attention over the past decade for its semiconducting properties (2.7 eV bandgap), excellent thermal and chemical stability, and, moreover, its promising photocatalytic activity.^31−35^ More importantly, g-C_3_N_4_ and α-Fe_2_O_3_ hybrids can be developed to present a band alignment that favors the formation of a direct Z-scheme structure upon adequate integration, rendering α-Fe_2_O_3_/g-C_3_N_4_ hybrids of great interest for various environmental photocatalytic applications.^36−39^ Conventional integration methods typically involve the solvothermal treatment of a g-C_3_N_4_ and iron oxide mixture^37,40,41^ or the calcination of an Fe-modified g-C_3_N_4_ precursor, such as melamine or urea.^42^ However, these approaches typically employ conventional thermal treatments carried out at high temperatures (of at least 550 °C) for long periods of time (beyond 2 h) to obtain the α-Fe_2_O_3_ phase. Apart from being a very energy-intensive process, this conventional thermal treatments also drastically restrict control over the morphology, size, and distribution of the resulting hematite nanoparticles on g-C_3_N_4_.^36,43−45^ By contrast, alternative technologies such as microwave reactor systems^46^ can both reduce the temperature required for this phase conversion to α-Fe_2_O_3_ while hastening the treatment process, features that are highly attractive toward the development of up-scalable photocatalytic systems with enhanced applicability.

Herein, we present an approach in which a novel two-step process overcomes limitations associated with the conventional fabrication of hematite-based Z-scheme photocatalytic systems. Our strategy involves an initial ultrasound-assisted impregnation of g-C_3_N_4_ with an aqueous FeCl_3_ precursor solution, followed by a short, mild microwave phase-conversion step, eliminating high-temperature thermal treatment methods. X-ray diffraction (XRD), thermogravimetric analysis (TGA), scanning transmission electron microscopy (STEM), and X-ray photoelectron spectroscopy (XPS) analyses provide a detailed picture of the overall growth mechanism of hematite (α-Fe_2_O_3_) nanoparticles on g-C_3_N_4_ sheets, emphasizing the critical role of the FeCl_3_ precursor concentration in achieving α-Fe_2_O_3_/g-C_3_N_4_ nanohybrids. Photo-Fenton degradation studies of methylene blue showcase their greatly boosted photocatalytic activity, which, owing to the intimate interaction between both components, is completely stable, as demonstrated by the subsequent recycling studies. The origin of this outstanding performance stems from the formation of an efficient Z-scheme heterojunction photocatalyst, as proven via spin-trapping electron spin resonance (ESR) experiments, Mott−Schottky measurements, and Tauc plot analysis.

## 2 Experimental Section

### 2.1 Materials and Chemicals

Iron(III) chloride (FeCl_3_, 97%, reagent grade) and melamine (99%) were purchased from Sigma-Aldrich and used in the preparation of hematite and carbon nitride, respectively. Methylene blue (MB, Panreac), rhodamine B (RhB, ≥95% (HPLC)), methyl red (MR, Panreac), and hydrogen peroxide (H_2_O_2_, 30% (v/v), analytical grade, Labkem) were used in the photocatalytic degradation experiments. Na_2_SO_4_ (ACS Reagent, ≥ 99.0%, anhydrous) was used as the electrolyte for the photoelectrochemical characterization. All the reagents used in this work were used without further purification.

### 2.2 Preparation of the Reference Materials

For the preparation of the akaganeite (β-FeOOH) nanoparticles, FeCl_3_ (1 M) was dissolved in Milli-Q water (10 mL) by magnetic stirring for 15 min until a dark yellow solution was obtained. This solution was then transferred to a glass reaction vessel and introduced into the microwave reactor (CEM Discover SP) and treated at 160 °C for 10 min. The power in the microwave reactor was set to 225 W to ensure a fast heating and a constant reaction temperature. A deep red solution with a precipitated solid was obtained, which was subsequently centrifuged at 10,000 rpm for 10 min to eliminate the supernatant. The remaining solid was dried at 75 °C overnight and ground to a fine brown powder, yielding the final β-FeOOH nanoparticles.

To obtain the hematite (α-Fe_2_O_3_) nanoparticles, β-FeOOH powder was put into a porcelain boat and placed in a horizontal tubular reactor. The akaganeite powder was then treated at 550 °C for 2 h with a heating ramp of 10 °C min^−1^. The as-obtained powder of hematite nanoparticles was employed as reference material in the characterization and photocatalytic control experiments.

The g-C_3_N_4_ powder was obtained by a commonly employed polycondensation method.^47^ In brief, 15 g of finely ground melamine was placed in a graphite crucible. This crucible was introduced into a muffle furnace and covered with a porcelain disk. The polycondensation process was carried out at 550 °C for 3 h with a heating ramp of 3 °C min^−1^. The as-obtained yellow powder was recovered from the crucible and ground to ensure easier dispersibility.

### 2.3 Preparation of the Nanohybrid Photocatalysts

The nanohybrid photocatalysts were prepared by a two-step process. In the first step (impregnation step), the ground g-C_3_N_4_ powder was dispersed (100 mg mL^−1^) in an aqueous solution of FeCl_3_ with varying concentrations ranging from 0.05 to 1 M with the help of an ultrasound probe (on−off cycle of 0.5 s at 50% over 90 min). The resulting dispersion was dried overnight at 75 °C, turning the characteristic yellow color of g-C_3_N_4_ into a reddish tone that indicated the presence of iron, and thus the successful impregnation of g-C_3_N_4_. In the second step (phase-conversion), the red-colored iron impregnated g-C_3_N_4_ powder was redispersed in 10 mL of Milli-Q water and introduced into a glass reaction vessel for a microwave treatment. The conditions of this microwave process were the same as those described above for the preparation of iron-based materials. After microwave treatment, a deep red solution with a precipitated solid was obtained. This solid was then recovered by centrifugation at 10000 rpm for 10 min, discarding the clear supernatant. Subsequently, the as-obtained powder was dried overnight at 75 °C and used without further manipulation.

### 2.4 Characterization of the Photocatalysts

The crystalline structure of the as-prepared photocatalyst powders was explored by X-ray diffraction (XRD) in a Bruker D-8 Advance diffractometer using Cu Kα as the radiation source (λ = 1.540 Å), from 5° to 80° with a step of 0.03° and an accumulation time of 5 s. The average crystalline domain size, lattice parameters, and phase composition were extracted through Rietveld refinement, using the integral width for the respective calculations. The thermal stability of the photocatalysts was investigated by thermogravimetric analysis (TGA), carried out in a Netzsch Libra F1 TGA system in an air atmosphere with a starting temperature of 30 °C, a final temperature of 800 °C, and a heating ramp of 10 °C min^−1^. The photoluminescence emission properties of the dispersed nanohybrid photocatalysts were studied using a Horiba Yvon FluoroMax-P, employing an excitation wavelength of 380 nm and a 10 mm path-length quartz cuvette. Similarly, UV−vis absorption spectra were collected by using a Shimadzu UV-2600 spectrophotometer. The morphology of the photocatalyst powders was characterized by scanning electron microscopy in a FEI INSPECT F-50 equipped with a field emission gun as an electron source. The size of the nanoparticles was measured using ImageJ, always using a representative number of features. Scanning transmission electron microscopy (STEM) and X-ray energy dispersive spectroscopy (XEDS) measurements have been performed using the Argonne PicoProbe Analytical Electron Microscope (AEM), which possesses the ANL XPAD system with its ultrasensitivity 4.5 sR detector.^48^ This microscope was operated at 300 kV. High-angle annular dark-field (HAADF)-STEM imaging was also performed using this instrument. X-ray photoelectron spectroscopy was collected in an ESCAPlus Omicron spectrometer equipped with an Al X-ray source (1486.7 eV, 300 W) and working at ultrahigh vacuum. For the high-resolution spectra, a step of 0.1 eV and a dwell time of 0.5 s were used. Spin-trapping Electron Spin Resonance (ESR) experiments were performed using 5,5-dimethyl-1-pyrroline N-oxide (DMPO) as a spin trap (Sigma-Aldrich, 97%). To this end, a stock solution of DMPO (500 mM) in H_2_O (Fischer, analytical grade) and methanol (Acros Organics, analytical grade) was prepared. Similarly, control hematite nanoparticles, pristine carbon nitride, and the α-Fe_2_O_3_/g-C_3_N_4_ 0.25 M nanohybrid (10 mg) were added to H_2_O or methanol (0.5 mL) and sonicated for 5 min in an ultrasound bath (BRANSONIC 1510E-MTH, 70 W, 42 kHz) to create the respective set of dispersions. Afterward, 50 μL of the stock DMPO solution was added to 50 μL of the 3 samples under investigation, resulting in a total concentration of 250 mM of DMPO. A small fraction of the dispersion was then transferred to a capillary (1 mm inner diameter) tube to measure the ESR spectra. UV-light from a Deuterium lamp was focused onto the samples placed in the ESR cavity, enabling ESR in situ measurements under illumination, starting immediately after light exposure. X-band continuous-wave ESR measurements were performed using a Bruker Elexsys-II E500 X-band spectrometer, operating at the X-band microwave frequency of 9.44 GHz. Samples were measured at room temperature in an ER4102ST rectangular TE102 cavity, with a microwave power of 50 mW and a modulation amplitude of 0.1 mT.

### 2.5 Photoelectrochemical Characterization

The photoelectrochemical measurements, including photocurrent response, electrochemical impedance spectroscopy, and Mott−Schottky analysis, were performed on an Autolab PGSTAT302N potentiostat using a LOT ORIEL solar simulator LS0106 as a light source, generating AM 1.5G light from a Xe Arc lamp (150 W) with a power density of 300 mW cm^−2^. A three-electrode setup was implemented with Na_2_SO_4_ (0.1 M) as the electrolyte, in which a Ag/AgCl (3 M KCl) electrode and a Pt wire acted as reference and counter electrodes, respectively. The working electrode was prepared by drop-casting the as-prepared photocatalysts dispersed in water (0.5 mg mL^−1^) on fluorine-doped tin oxide (10 mm × 25 mm, TEC-15, Pilkington), followed by drying at 150 °C for 1 h. The equivalent circuit modeling and impedance data evaluation were performed using the NOVA software.

### 2.6 Photocatalytic Degradation Studies

The photocatalytic activity of the reference materials and α-Fe_2_O_3_/g-C_3_N_4_ nanohybrids was explored by room-temperature degradation of methylene blue (MB) through a photo-Fenton process. In a typical experiment, an aqueous solution of MB (15 mg L^−1^) was prepared. For each experiment, 15 mL of this solution was introduced into a closed-cap vial along with 7.5 mg of the desired photocatalyst and stirred in dark conditions for 1 h to ensure the adsorption/desorption equilibrium between photocatalyst and contaminant was achieved. Then, 1 mL of H_2_O_2_ (0.02% v/v) was added to the MB solution to act as a co-oxidant, and the vial was exposed to UV light (365 nm, from a TL 8W Philips lamp), denoting the beginning of the experiment. To track the photocatalytic activity, the degradation of the MB dye was followed by measuring the absorbance of the solution at 664 nm (wavelength at which MB exhibits its maximum absorption) employing a Shimadzu UV-2600 spectrophotometer, specifically at 664 nm, which corresponds to the wavelength of maximum absorption of MB. The first aliquot of MB was taken before the addition of H_2_O_2_, the second one (*t* = 0 min) was taken after the addition of the H_2_O_2_, and then every 10 min thereafter. The degradation of MB was considered a pseudo-first-order reaction, and the kinetic model employed is described in eq 1:
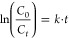
1where *C*_0_ is the concentration at *t* = 0 min, *C_t_* is the concentration at time *t*, and *k* is the kinetic constant of the pseudo-first order, obtained from the slope of the degradation curve represented according eq 1. An example of the evolution of the degradation is included in the Supporting Information, see Figure S11. To assess the applicability of the as-prepared photocatalysts to other pollutant systems, the degradation of rhodamine B (RhB) and methyl red (MR) was studied in the same manner as MB. Their degradation was thus tracked by following their maximum absorption, achieved at 553 nm for RhB and at 524 nm for MR, respectively.

## 3 Results and Discussion

### 3.1 Characterization of α-Fe_2_O_3_/g-C_3_N_4_ Nanohybrid Photocatalysts

**Scheme 1:**
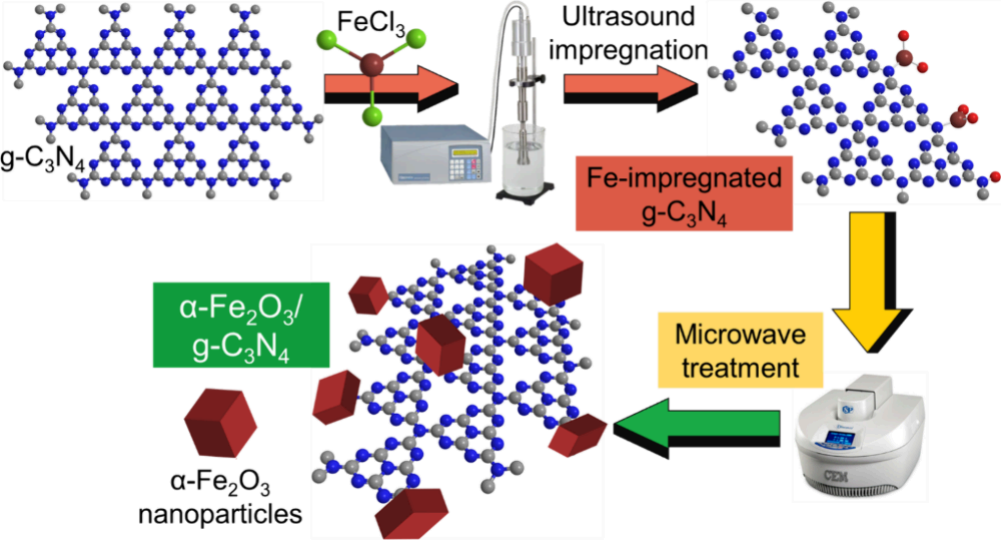
Representation of the Two-Step Preparation of the α-Fe_2_O_3_/g-C_3_N_4_ Nanohybrids, Initiated by an Ultrasound Impregnation of g-C_3_N_4_ with an Aqueous FeCl_3_ Precursor Solution, followed by a Microwave Treatment That Induces the Phase-Conversion of the Anchored Fe Species into α-Fe_2_O_3_ Nanoparticles

The preparation of the α-Fe_2_O_3_/g-C_3_N_4_ nanohybrid photocatalysts followed a two-step process. It is initiated by an ultrasound-assisted impregnation of pristine g-C_3_N_4_ sheets with an aqueous solution of FeCl_3_, followed by a mild and short microwave-driven phase-conversion step, in which the anchored iron precursor species are converted into hematite nanoparticles that are well integrated onto g-C_3_N_4_ sheets. Scheme 1 outlines the complete preparation pathway, while the specific details on the synthesis conditions of all reference and nanohybrid materials are fully described in Section 2.

**Figure 1:**
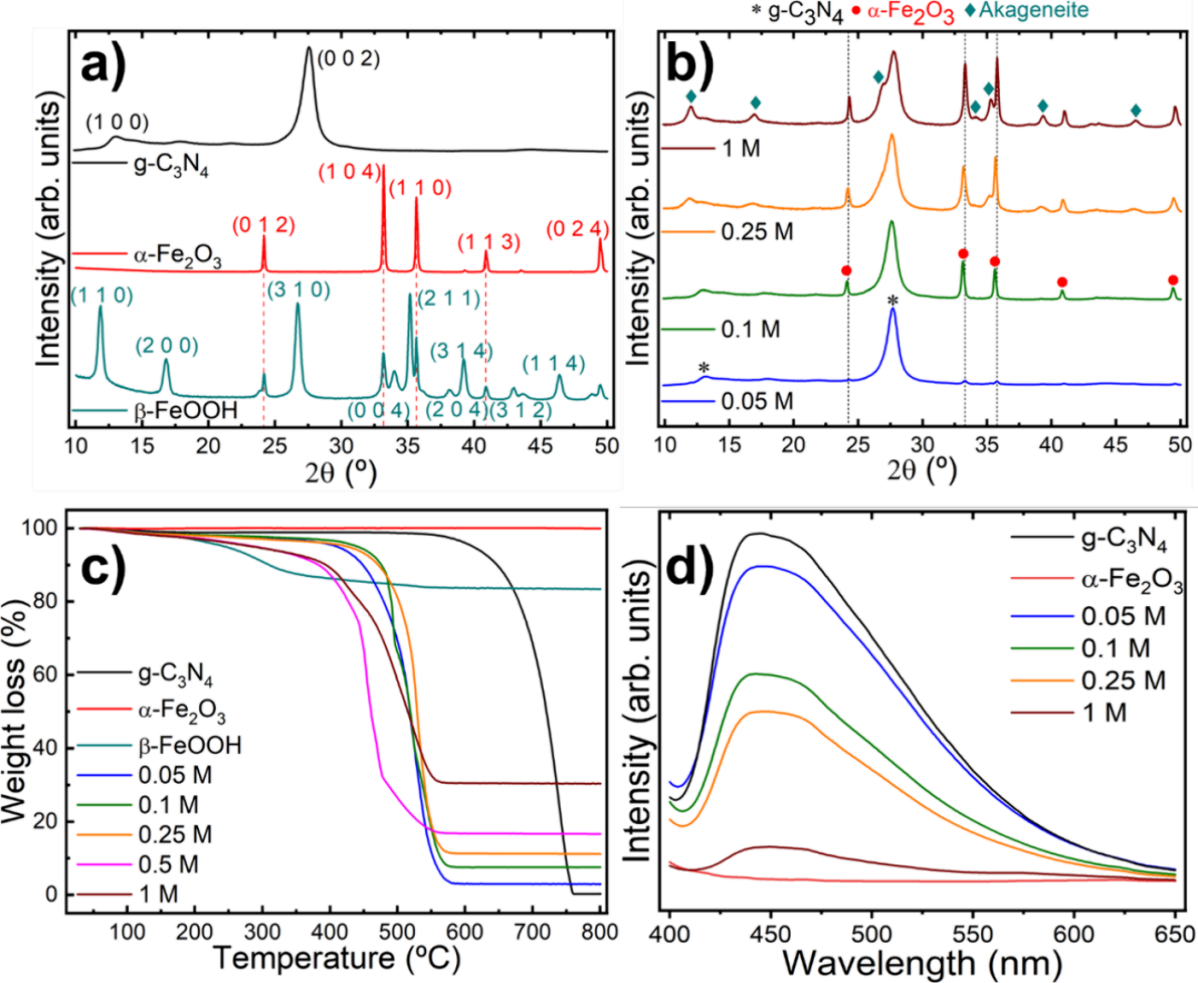
X-ray diffraction pattern of (a) g-C_3_N_4_, β-FeOOH, and α-Fe_2_O_3_ nanoparticles and (b) α-Fe_2_O_3_/g-C_3_N_4_ nanohybrid photocatalysts, as a function of the FeCl_3_ concentration used during the impregnation process. (c) Thermogravimetric analysis and (d) photoluminescence spectroscopy of the as-prepared photocatalysts.

The XRD pattern of akaganeite nanoparticles used as a reference sample (Figure 1a, black line) shows β-FeOOH as the main crystal phase, identified by its prominent (1 1 0), (3 1 0), and (2 1 1) peaks (see JCPDS Card Number 34-1266). Additionally, diffraction peaks corresponding to α-Fe_2_O_3_ are detected (highlighted by the red dotted lines), yielding a composition of 36.1% α-Fe_2_O_3_ and 63.9% β-FeOOH as determined by Rietveld refinement (Table S1). Subsequent thermal treatment at 550 °C for 2 h transforms akaganeite (β-FeOOH) nanoparticles into pure hematite phase (Figure 1a, red line),^49^ with well-defined (0 1 2), (1 0 4), (1 1 0) and (1 1 3) components (see JCPDS Card Number 33-0664). Lattice parameters and crystalline domain sizes are also detailed in Table S1 in the Supporting Information. The diffractogram of pristine g-C_3_N_4_ displays the two characteristic diffraction peaks at 13.0° (1 0 0) and 27.5° (0 0 2), related to the in-plane intralayer *d*-spacing and the interlayer distance of the conjugated aromatic ring system, respectively (see JCPDS Card Number 87-1526).^50^

The XRD analysis of the nanohybrid materials (Figure 1b) reveals a significant influence of the concentration of the FeCl_3_ precursor used in the impregnation step. At the lowest FeCl_3_ concentration (0.05 M), the diffraction peaks at 13.0° and 27.6° belonging to g-C_3_N_4_ dominate the diffractogram (black dotted lines), accompanied by weak signals from α-Fe_2_O_3_ (red dashed lines). Increasing the FeCl_3_ concentration to 0.1 M leads to significantly more prominent hematite peaks. This prominence rises until a threshold concentration of 0.25 M is reached, at which point the slight emergence of the β-FeOOH akaganeite phase is denoted, while maintaining the g-C_3_N_4_ contribution constant. Increasing the FeCl_3_ concentration to 1 M furthers the formation of β-FeOOH, as evidenced by the clear presence of the (1 1 0), (3 1 0) and (2 1 1) akaganeite diffraction peaks that entail a relative decrease in the intensity of the hematite peaks with respect to g-C_3_N_4_ (see Table S1). Moreover, there is a correlation between this increasing FeCl_3_ concentration and the widening and reduction in intensity of the intraplanar (1 0 0) peak of g-C_3_N_4_ in the nanohybrid materials (see Figure S1). This trend suggests that higher FeCl_3_ concentrations during impregnation lead to smaller and more defective g-C_3_N_4_ sheets, likely due to the more acidic conditions of the process.^51^ Moreover, the presence of akaganeite at high FeCl_3_ concentrations suggests the existence of a concentration threshold at around 0.25 M at which the hematite content is maximized along with minimal akaganeite.

It should be noted that akaganeite is a well-known product of the synthesis of FeCl_3_ that, through thermal treatment at temperatures above 550 °C, is converted into hematite. However, in this work, this phase conversion is achieved during the short and mild microwave reactor treatment, without requiring further thermal treatment, evidencing the active and catalytic role of the g-C_3_N_4_ surface in this phase-conversion process. These XRD results clearly highlight the efficacy of the two-step synthetic process in promoting the controlled incorporation of α-Fe_2_O_3_ nanoparticles onto g-C_3_N_4_, facilitated by the catalytic effect of g-C_3_N_4_ sheets during the microwave-driven phase-conversion process of β-FeOOH into α-Fe_2_O_3_.

Thermogravimetric analysis under air provides insight into the loading of α-Fe_2_O_3_ nanoparticles grown in the nanohybrid photocatalysts and their critical dependency on the precursor concentration (Figure 1c). Pristine g-C_3_N_4_ exhibits thermal stability up to 570 °C, followed by a pronounced weight loss, resulting in near-complete removal of the starting material with only 0.36 wt % remaining. By contrast, β-FeOOH initially shows a modest weight loss beginning at a significantly lower temperature (100 °C), gradually progressing until 600 °C, and retaining 83.3% of its initial weight. This weight loss is attributed to the phase conversion from β-FeOOH to α-Fe_2_O_3_ as a result of the oxidation of akaganeite. Conversely, α-Fe_2_O_3_ nanoparticles reveal high thermal stability without any apparent weight loss up to the maximum experimental temperature probed (800 °C). Consequently, the thermal stability of the nanohybrid photocatalysts depends on the iron oxide phase and its total iron content. This interplay between precursor concentration and thermal stability becomes particularly evident in nanohybrids prepared with lower FeCl_3_ concentrations (0.05, 0.1, and 0.25 M). These nanohybrids that contain a pure hematite phase, as demonstrated by the XRD results, exhibit a weight loss behavior intermediate between pristine g-C_3_N_4_ and α-Fe_2_O_3_ nanoparticles. This is characterized by a single weight loss, attributed to the decomposition of carbon nitride, which commences at around 420 °C. Consequently, the residual mass directly correlates with the iron oxide concentration, yielding values of 2.95, 7.54, and 11.16 wt % for the 0.05, 0.1, and 0.25 M precursor concentrations, respectively. In addition, the degradation temperature in these nanohybrids is significantly lower compared to pristine g-C_3_N_4_, a phenomenon likely caused by the presence of iron oxide nanoparticles integrated onto the g-C_3_N_4_ sheets, known to catalyze the thermal decomposition of carbon-based materials.^52,53^ Conversely, hybrids prepared with higher iron precursor concentrations (0.5 and 1 M) display a marked decrease in thermal stability since their weight loss initiates already at around 150 °C. The origin behind this diminished stability is likely due to the more prominent presence of the β-FeOOH phase, which features a much lower thermal stability compared to both α-Fe_2_O_3_ and g-C_3_N_4_ at low temperatures. In addition, and in agreement with XRD findings (Figure 1b), the 0.5 and 1 M hybrids reveal a higher iron content, with residual values of 16.65 and 30.40 wt %, respectively. These results underscore the critical role of a pure α-Fe_2_O_3_ phase in ensuring the thermal stability of the hybrid photocatalyst, with the 0.25 M precursor concentration serving as a critical threshold.

The photoluminescence (PL) spectrum of the α-Fe_2_O_3_/g-C_3_N_4_ photocatalysts is shown in Figure 1d. Pristine carbon nitride exhibits a wide emission band with a maximum at 445 nm for an excitation wavelength of 380 nm, which corresponds with π → π* transitions of g-C_3_N_4_ (corresponding UV−vis spectra are depicted in Figure S2 of the Supporting Information). Conversely, the as-prepared α-Fe_2_O_3_ nanoparticles did not show any photoluminescent behavior. Thus, the hematite content can be easily tracked via PL by its effect on the emissive properties of g-C_3_N_4_: larger α-Fe_2_O_3_ contents stemming from higher FeCl_3_ concentrations during the impregnation step lead to dwindling emission signal, which is the largest for the 0.05 M nanohybrid and minimal for the 1 M. This effect can be directly correlated with the successful integration of α-Fe_2_O_3_ and g-C_3_N_4_, leading to a decrease in the radiative recombination of charge carriers and its resulting enhancement in the charge separation and transfer for the nanohybrid photocatalysts.^54^

**Figure 2:**
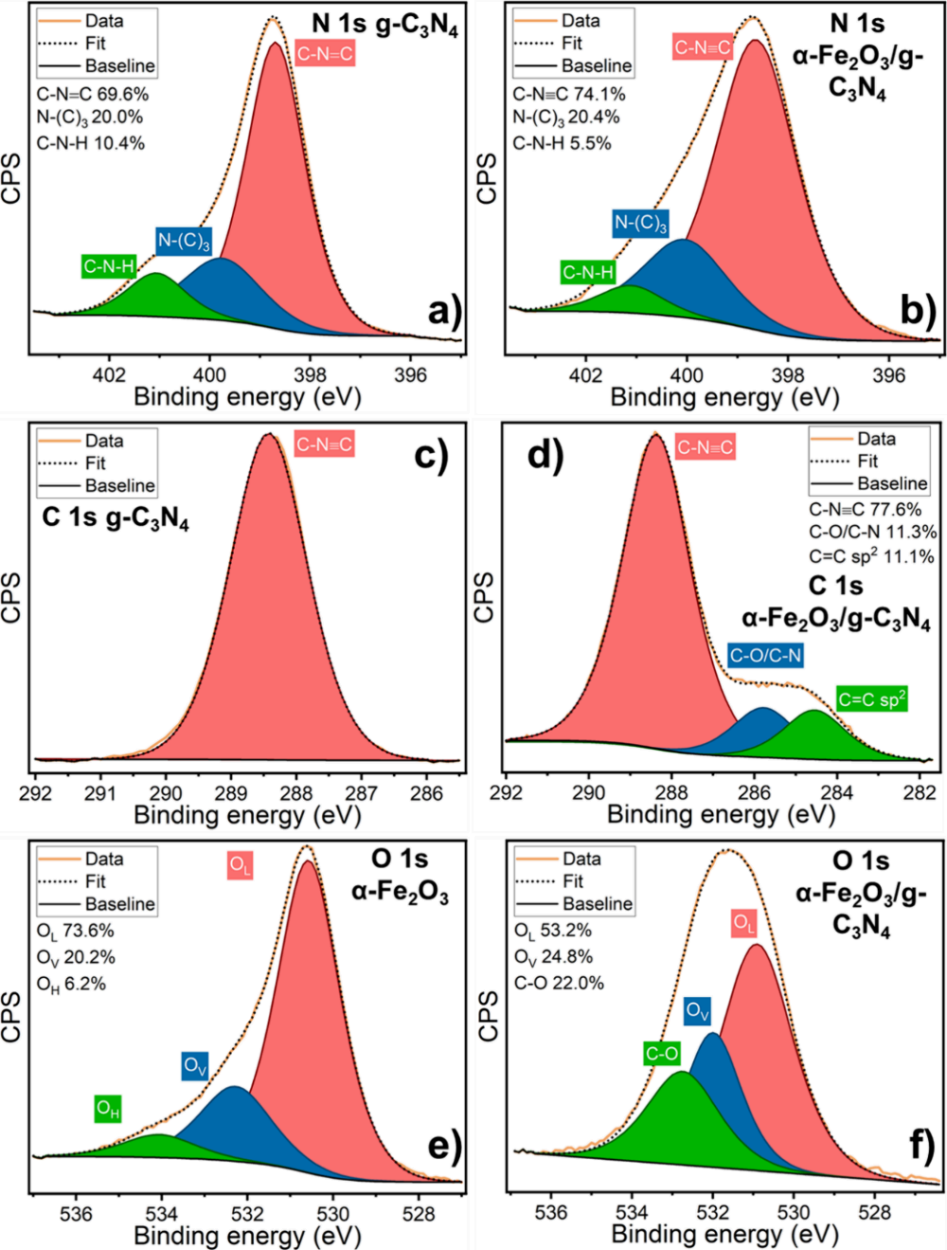
XPS core spectra of g-C_3_N_4_, α-Fe_2_O_3_, and α-Fe_2_O_3_/g-C_3_N_4_ 0.25 M and the deconvolution of their (a, b) N 1s, (c) and (d) C 1s, (e), and (f) O 1s contributions.

Since the intimate interaction between g-C_3_N_4_ and α-Fe_2_O_3_ has been clearly demonstrated, high-resolution X-ray photoelectron spectroscopy (N 1s, C 1s, and O 1s) was utilized to study the evolution of the surface chemistry of α-Fe_2_O_3_ nanoparticles and g-C_3_N_4_ upon the formation of the nanohybrid. The deconvoluted N 1s core spectra of g-C_3_N_4_ reveals three different contributions^55^ (Figure 2a): a main peak at 398.7 eV, corresponding to structural sp^2^ N (C−N=C) of the heptazine rings, a secondary contribution at 399.8 eV ascribed to tertiary nitrogen (N−(C)_3_), and a minor peak at 401.1 eV that confirms the presence of amino groups. By contrast, the N 1s peak of the α-Fe_2_O_3_/g-C_3_N_4_ 0.25 M nanohybrid (Figure 2b) exhibits substantial differences, although still retaining these components. Notably, the shoulder observed at 401 eV for g-C_3_N_4_ displays a significant decrease in the nanohybrid, indicating the diminished presence of free amino groups. This correlates with the reduction in the contribution of this component from 10.4 to 5.5% and a corresponding increase of the sp^2^ N component from 69.6 to 74.1%. This change is ascribed to the cleavage of the g-C3N4 sheets observed in XRD and suggests the partial substitution of the amino terminal for other stable bonds such as Fe−N.^56,57^ The C 1s core spectra of g-C_3_N_4_ (Figure 2c) display two contributions corresponding to the C−N=C component at 288.4 eV and the C−C component, centered at 282.7 eV, related to the sp^2^ C−C bond (adventitious or derived from unreacted melamine).^58−60^ Conversely, the C 1s peak of the nanohybrid material (Figure 2d) revealed significant alterations. While the C−N=C component at 288.4 eV remains the primary contribution, two new signals emerge at lower binding energies, evidenced by the broad shoulder centered at 285 eV. Its two contributions, located at 285.8 and 284.6 eV, are ascribed to the C−N/C−O bonding and C=C/C−C carbon bonds, respectively. The former C−N/C−O component likely indicates the integration of α-Fe_2_O_3_ onto g-C_3_N_4_, potentially involving Fe−N or O−C bonds. The latter C=C/C−C component suggests the presence of defects within the g-C_3_N_4_ structure^61,62^ originating from the breakup of the g-C_3_N_4_ sheets during the ultrasound probe treatment, as discussed in the context of the XRD results (see also Figure S1 in Supporting Information). XPS analysis further reveals the significant changes observed in the chemical environment of α-Fe_2_O_3_. The O 1s peak of the as-prepared α-Fe_2_O_3_ nanoparticles (Figure 2e) reveals three distinct contributions. The first, located at 530.6 eV, is ascribed to the structural lattice oxygen of α-Fe_2_O_3_ and represents the most significant component (73.6%). The second contribution at 532.3 eV corresponds to oxygen vacancies characteristic of the hematite surface structure, accounting for 20.2% of the total oxygen content.^63^ Finally, the third component, located at 534.1 eV, is related to water and other oxygen-related species adsorbed on the surface of the nanoparticles. In comparison, the O 1s peak of the α-Fe_2_O_3_/g-C_3_N_4_ nanohybrid (Figure 2f) exhibits a broader profile, divided into three components: the main one at 530.9 eV corresponds to lattice oxygen (53.2%); the second contribution, centered at 532.1 eV, is attributed to the oxygen vacancies of α-Fe_2_O_3_ and accounts for 24.8% of the oxygen content; and the third component, located at 532.8 eV, is ascribed to the C−O contribution, in agreement with the C 1s observations discussed above. An eventual contribution of oxygen species adsorbed on hematite is not discernible. The clear change in the O 1s peak shape from α-Fe_2_O_3_ to the nanohybrid and the appearance of a substantial C−O contribution both point toward a drastic modification of the chemical environment of α-Fe_2_O_3_ upon integration with carbon nitride, in accordance with the results from the N 1s and C 1s XPS spectra. The Fe 2p data for both α-Fe_2_O_3_ and the α-Fe_2_O_3_/g-C_3_N_4_ 0.25 M nanohybrid (Figure S3, Supporting Information) further corroborate these chemical modifications. Moreover, these results derived from Figures 1 and 2 assisted in the development of the cleavage and impregnation mechanism that g-C_3_N_4_ undergoes during the ultrasound impregnation, as illustrated in Figure S4.

**Figure 3:**
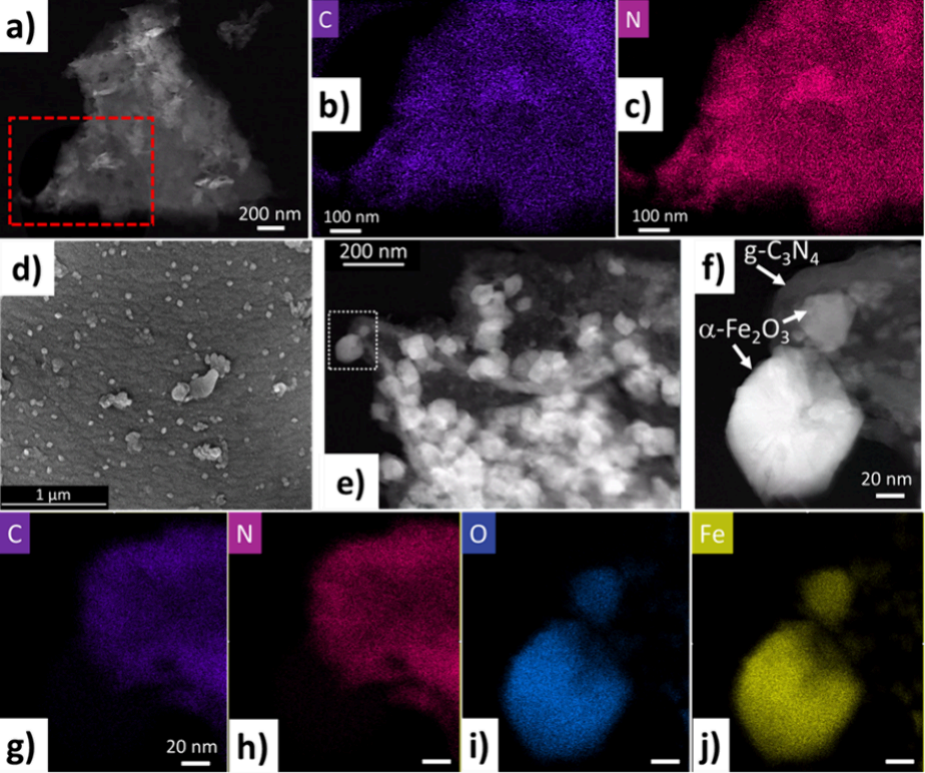
(a) High-angle annular dark-field image (HAADF-STEM) of pristine g-C_3_N_4_. (b) Carbon and (c) nitrogen STEM-XEDS elemental maps recorded in the area highlighted in red in Figure 3a. (d) FESEM micrograph of the 0.25 M α-Fe_2_O_3_/g-C_3_N_4_ nanohybrid photocatalyst. (e) Low and (f) high magnification HAADF-STEM images of said hybrid, along with the (g) carbon, (h) nitrogen, (i) oxygen, and (j) iron STEM-XEDS elemental maps of the α-Fe_2_O_3_/g-C_3_N_4_ 0.25 M nanohybrid.

Morphological, structural, and elemental analyses of pristine g-C_3_N_4_, α-Fe_2_O_3_ nanoparticles, and α-Fe_2_O_3_/g-C_3_N_4_ hybrids were performed by scanning transmission electron microscopy (STEM), imaging, and energy dispersive X-ray spectroscopy (EDX), as well as field-emission scanning electron microscopy (FESEM). Pristine g-C_3_N_4_ flakes exhibit a planar morphology of stacked sheets (Figure S5a). These flakes, examined by STEM high-angle annular dark-field (HAADF) imaging and STEM EDX (Figure 3a−c), reveal a homogeneous elemental colocalization of carbon and nitrogen across the entire g-C_3_N_4_ sheet. FESEM analysis of the as-prepared α-Fe_2_O_3_ nanoparticles displays a unique planar platelet morphology (Figure S5b, Supporting Information) with an average particle size of about 200 nm. However, α-Fe_2_O_3_ nanoparticles integrated with the α-Fe_2_O_3_/g-C_3_N_4_ 0.25 M nanohybrid (Figure 3d), adopt a distinct polyhedral hematite morphology with an average size of about 165 nm (Figure S5e, Supporting Information). In addition, FESEM investigations of the remaining hybrids (Figure S5c−f, Supporting Information) show that an increasing FeCl_3_ impregnation concentration correlates with an enlargement of the size of the α-Fe_2_O_3_ nanoparticles. These larger nanoparticles most likely originate from the aggregation and coalescence of smaller α-Fe_2_O_3_ nanoparticles, eventually leading to complete coverage of the g-C_3_N_4_ flake surface. HAADF-STEM analysis of the α-Fe_2_O_3_/g-C_3_N_4_ 0.25 M nanohybrid (Figure 3e,f) corroborates the presence of both the polyhedral α-Fe_2_O_3_ nanoparticles and these smaller nanoparticles residing on the surface of g-C_3_N_4_ (Figure 3f). The elemental mapping for carbon and nitrogen elemental maps (Figure 3g,h) confirms a clear spatial colocalization of these elements, as observed for the pristine g-C_3_N_4_ (Figure 3b,c). Notably, no C or N signal is detected in the region corresponding to the larger α-Fe_2_O_3_ nanoparticles. These findings provide evidence that the bright polyhedral spots observed in Figure 3e, f exclusively represent the α-Fe_2_O_3_ nanoparticles directly grown onto the g-C_3_N_4_ surface, which correlates with the appearance of iron and oxygen signals from these nanoparticles (Figure 3i, j).

### 3.2 Assessment of the Photocatalytic Activity of the α-Fe_2_O_3_/g-C_3_N_4_ Photocatalysts

**Figure 4:**
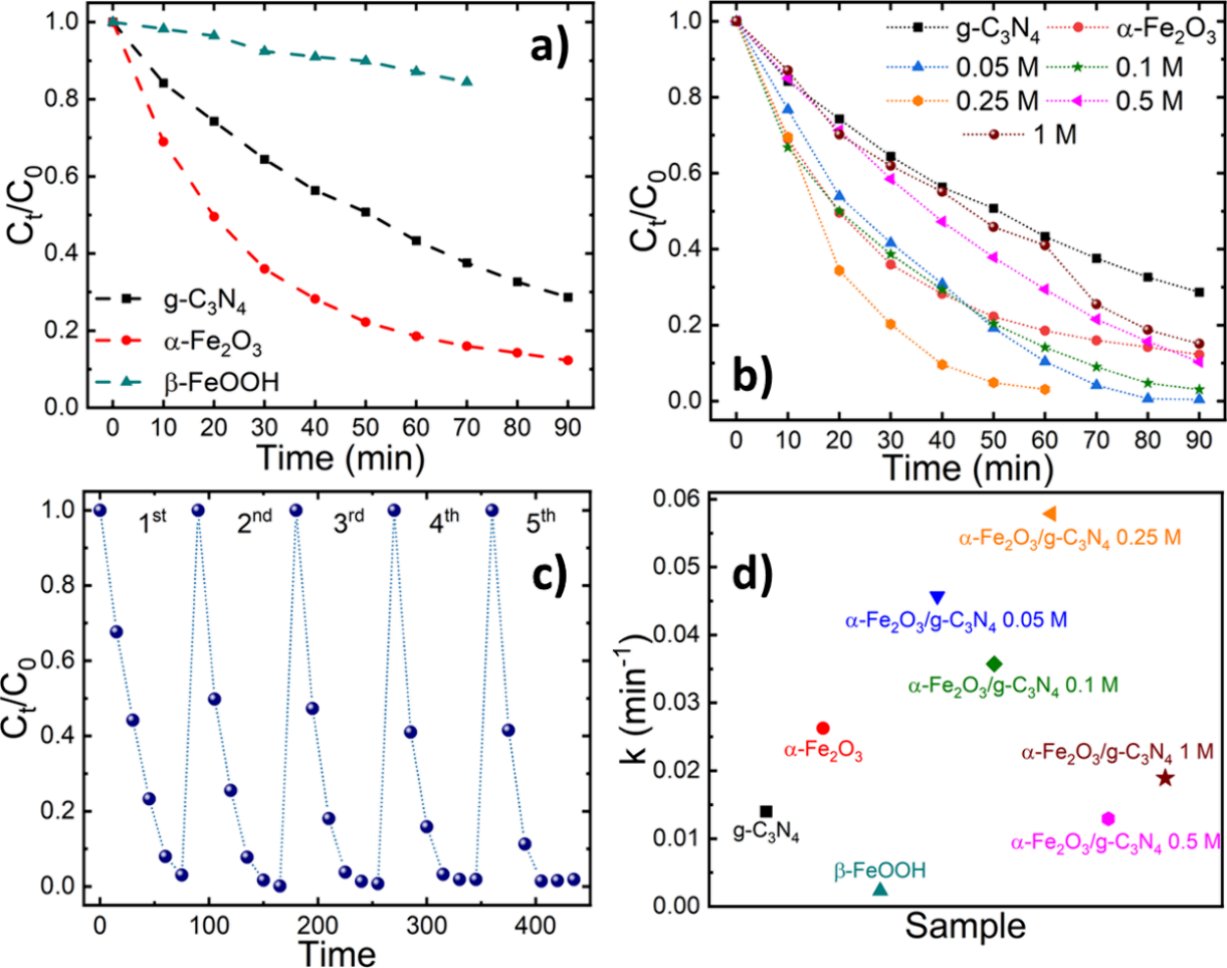
Evolution of the degradation of methylene blue solutions in the presence of (a) g-C_3_N_4_, β-FeOOH, and α-Fe_2_O_3_ nanoparticles, (b) nanohybrid photocatalysts prepared with varying FeCl_3_ concentrations from 0.05 to 1 M. (c) Recycling study of the best performing photocatalyst. (d) Comparison of the estimated pseudo-first order kinetic constants for the degradation of MB.

The photocatalytic activity of the as-prepared nanohybrid photocatalysts was explored following the degradation of methylene blue (MB) in a photo-Fenton process (see Section 2 for details). Figure 4a depicts the comparative photocatalytic activity of g-C_3_N_4_, α-Fe_2_O_3_, and β-FeOOH. The hematite formation intermediate, β-FeOOH, yields the lowest activity, achieving a mere 19% degradation of the initial MB concentration after 70 min. Conversely, g-C_3_N_4_ and α-Fe_2_O_3_ nanoparticles show significantly higher activity, reducing the initial MB concentration to 29 and 12%, respectively, after a 90-min degradation period. These findings suggest that higher concentrations of β-FeOOH correlate with lower photocatalytic activity, while a higher α-Fe_2_O_3_ content translates to an enhanced MB degradation efficiency. Figure 4b illustrates the photocatalytic activity of the nanohybrid photocatalysts prepared with varying FeCl_3_ precursor concentrations. Notably, the nanohybrid prepared with a 0.25 M precursor concentration outperforms both reference materials and other hybrids, achieving a complete dye degradation in just 60 min. Nanohybrids prepared with lower FeCl_3_ concentrations (0.05 and 0.1 M) exhibit moderate performance, completely removing MB in 80 min. While not as efficient as the 0.25 M photocatalyst, their activity remains superior to the reference materials. By contrast, catalysts prepared with higher FeCl_3_ concentrations (0.5 and 1 M) demonstrate poorer performance, failing to completely degrade MB even after 90 min. These results highlight the detrimental impact that a high β-FeOOH content has on the photocatalytic activity of the nanohybrids, emphasizing the critical importance of preparing iron oxide photocatalysts with a high degree of hematite phase-purity. Thus, the 0.25 M FeCl_3_ concentration represents a critical threshold value for the optimal photocatalytic activity. At this concentration, the nanohybrids achieve the highest α-Fe_2_O_3_ content while minimizing the β-FeOOH phase. Moreover, as discussed above, this FeCl_3_ precursor concentration also promotes a decrease in the planar size of the g-C_3_N_4_ sheets, which in turn enhances the available catalytic surface area. Complementary to this study, the need for both H_2_O_2_ as co-oxidant and UV light irradiation (and its comparison with visible light illumination) for the degradation of MB was demonstrated for α-Fe_2_O_3_, g-C_3_N_4_, and the α-Fe_2_O_3_/g-C_3_N_4_ 0.25 M nanohybrid, see Figures S6 and S7. Importantly, additional experiments examining the concentration dependency of this nanohybrid (Figure S8) reveal that reducing the photocatalyst concentration by up to one-third yields degradation rates still faster than those obtained compared to any of the other studied photocatalysts. Finally, degradation studies of two other organic dyes (rhodamine B, RhB, and methyl red, MR, see Figure S9) further demonstrate the efficacy of this α-Fe_2_O_3_/g-C_3_N_4_ in the photocatalytic removal of different organic pollutants.

To assess the stability of the best-performing photocatalyst, the α-Fe_2_O_3_/g-C_3_N_4_ 0.25 M nanohybrid, a recycling study was carried out. Hereto, the degradation of MB was consecutively repeated five times, employing the photocatalyst recovered after each experiment. Importantly, no mass of photocatalyst was appreciated throughout the entire recycling experiment, ensuring that all degradation experiments were carried out under the same conditions. Figure 4c shows that the photocatalyst was completely stable through the five consecutive experiments, obtaining the complete degradation of MB in 60 min in all five experiments. Interestingly, the degradation rate is slightly improved after the first experiment since the complete removal of MB is achieved at shorter times for the later cycles. This suggests a possible activation of the photocatalyst as the origin of this enhancement. Furthermore, XRD analysis clearly confirms that the recovered photocatalyst did not suffer any structural changes upon the cycling experiments, thus highlighting its stability (see Supporting Information Figure S10)

Figure 4d compares the estimated reaction constants for all investigated photocatalysts, assuming a pseudo-first-order photo-Fenton degradation process of MB, as described in the Supporting Information. Among the reference materials, α-Fe_2_O_3_ exhibits the highest reaction rate constant (0.026 min^−1^), significantly exceeding those of g-C_3_N_4_ (0.014 min^−1^) and β-FeOOH (0.002 min^−1^), as expected for a photo-Fenton process. Moreover, the kinetic constant of hematite surpasses those of the 0.5 and 1 M hybrids, which displayed values of 0.013 and 0.019 min^−1^, respectively. In contrast, the low concentration photocatalysts (0.05, 0.1, and 0.25 M) outperform hematite, revealing reaction rate constants of 0.046, 0.036, and 0.058 min^−1^, respectively. The 0.25 M nanohybrid thus exhibits the highest rate constant of all photocatalysts studied. Its overall performance and stability are comparable or superior to other related material systems, see Table S2 in the Supporting Information.

### 3.3 Empirical Evidence for the Photocatalytic Mechanism

**Figure 5:**
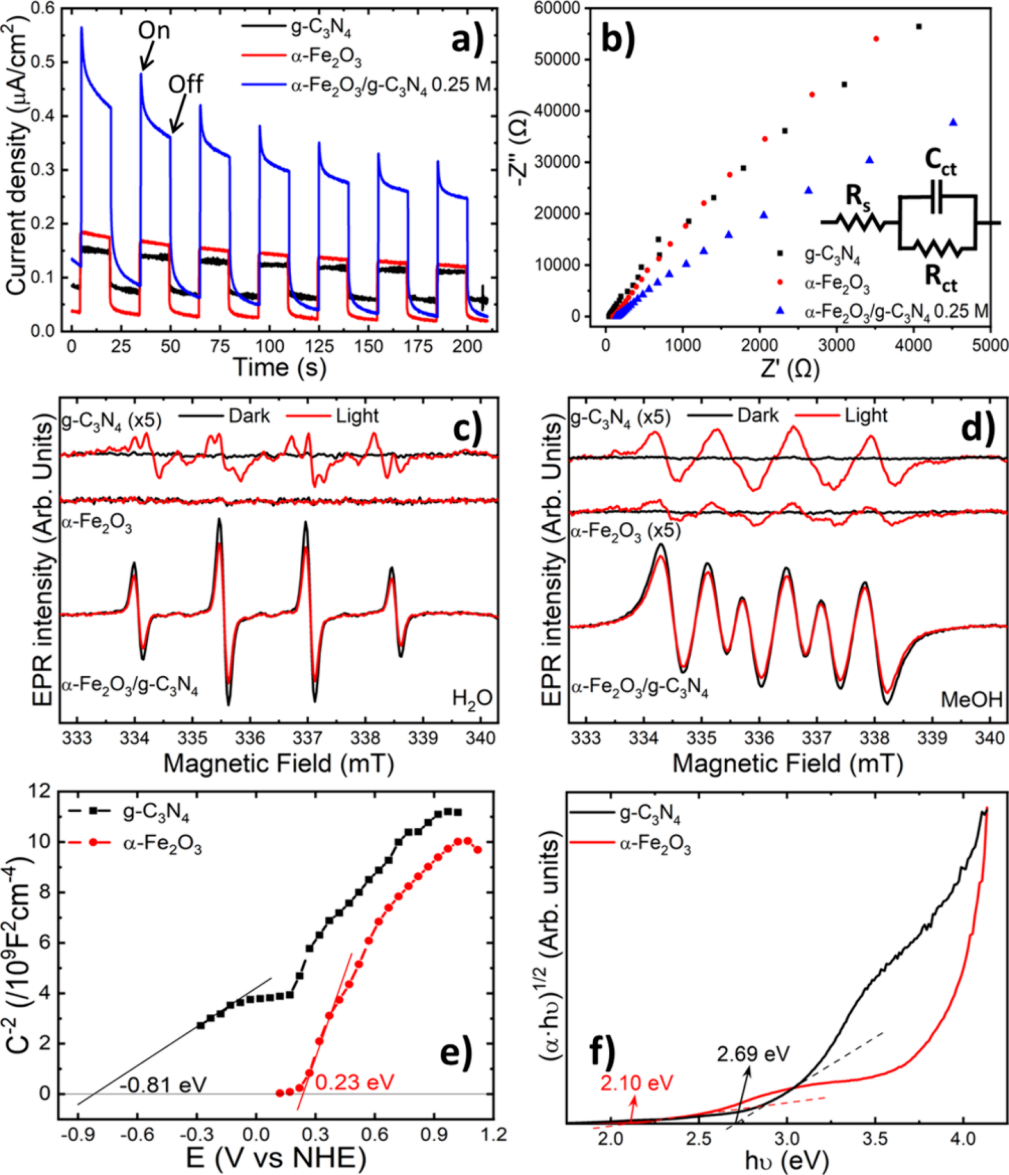
(a) Chronoamperometry, (b) Nyquist diagram, (c) DMPO-·OH, and (d) DMPO-·O_2_^−^ measurements of g-C_3_N_4_, α-Fe_2_O_3_, and α-Fe_2_O_3_/g-C_3_N_4_ nanohybrids. (e) Mott−Schottky and (f) Tauc plots of g-C_3_N_4_ and α-Fe_2_O_3_.

The significantly enhanced performance of the α-Fe_2_O_3_/g-C_3_N_4_ nanohybrid underscores the efficacy of the combined impregnation and microwave treatment in producing a superior nanohybrid photocatalyst with considerably boosted photocatalytic activity that is attributed to the direct growth of α-Fe_2_O_3_ nanoparticles on the surface of the g-C_3_N_4_ sheets. In the next step, the origin of the enhanced photocatalytic performance of the nanohybrid photocatalysts and their underlying electronic working mechanism are examined. First, chronoamperometric measurements were carried out to determine the photoresponse of the photocatalysts. Results presented in Figure 5a show that g-C_3_N_4_ yields the lowest photocurrent, closely followed by that of α-Fe_2_O_3_. In comparison, the α-Fe_2_O_3_/g-C_3_N_4_ nanohybrid photocatalyst exhibits a photocurrent more than two times higher than that of the base materials, indicating an improved efficiency in the separation and transfer of photogenerated electrons and holes in the nanohybrid, even when considering its transitory decrease during the ON cycle. Electrochemical impedance spectroscopy (EIS) measurements further confirm the improved charge separation and transfer capabilities of the α-Fe_2_O_3_/g-C_3_N_4_ nanohybrid. The Nyquist diagram arc of this nanohybrid photocatalyst (Figure 5b) is considerably smaller than those of g-C_3_N_4_ and α-Fe_2_O_3_. This translates into lower charge transfer resistance (*R*_ct_) and capacitance (*C*_ct_) for the nanohybrid photocatalyst according to the equivalent circuit indicated in Figure 5b, thus clearly evidencing its faster charge separation and transfer rate capability.

To corroborate the performance of the α-Fe_2_O_3_/g-C_3_N_4_ nanohybrid, spin-trapping electron spin resonance experiments were conducted. 5,5-dimethyl-1-pyrroline N-oxide (DMPO) was employed as a commonly used spin trap known for stabilizing oxygen-containing short-lived radicals.^64,65^ Thus, DMPO was added in water and methanol dispersions containing the control and nanohybrid photocatalysts to investigate the presence of hydroxyl (•OH) and superoxide (•O_2_^−^) radicals, respectively, the oxygen reactive species that drive the photodegradation of MB. Figure 5c illustrates the ESR spectra of α-Fe_2_O_3_, g-C_3_N_4_, and the α-Fe_2_O_3_/g-C_3_N_4_ 0.25 M nanohybrid photocatalysts dispersed in H_2_O after adding DMPO, both in the dark and under UV illumination. Consistent with their photocatalytic behavior, the reference materials exhibit no detectable ESR signal in the dark. Interestingly, even under UV-illumination, α-Fe_2_O_3_ nanoparticles still do not show any •OH formation, contrasting with the weak signal observed for g-C_3_N_4_. The g-C_3_N_4_ spectrum exhibits the characteristic line shape and hyperfine splitting pattern associated with the DMPO-•OOH spin adduct. Notably, the nanohybrid displays a clear ESR signal with the typical 4-line pattern and a 1:2:2:1 intensity ratio characteristic of DMPO-•OH,^64,66^ indicating the strong formation of hydroxyl radicals.

Figure 5d presents the ESR spectra obtained in a methanol solution, which was used to detect the presence of the DMPO-•O_2_^−^ radical. In dark conditions, the reference materials did not show any signal. However, upon UV illumination, the •O_2_^−^ radical is detected in both control photocatalysts, with g-C_3_N_4_ exhibiting a higher sensitivity compared to α-Fe_2_O_3_. Interestingly, the nanohybrid shows a significantly more intense signal, both in dark conditions and under UV illumination, consistent with previous findings ascribing this signal to the presence of •O_2_^−^ radicals.^67,68^ In addition, a decrease in this signal intensity is observed under UV illumination, mirroring the behavior of the DMPO-•OH signal. These results clearly highlight the enhanced ability of the nanohybrid photocatalyst in generating a significantly higher number of radicals compared to the reference materials.

In order to understand the mechanism governing the superior charge transfer characteristics, the enhanced formation of radicals, and photocatalytic degradation properties, the electronic properties of the nanohybrid heterojunction were examined. The band structure and band alignment of its components were empirically determined by Mott−Schottky measurements and Tauc plot analyses. The negative slope of the Mott−Schottky curves represented in Figure 5e reveals the typical n-type semiconductor behavior of both materials, with flat-band potentials of 0.81 and 0.23 V (vs the NHE) for g-C_3_N_4_ and α-Fe_2_O_3_, respectively. Finally, the Tauc plot analysis obtained from the UV−vis measurements in Figure S2a reveals bandgap values of 2.69 and 2.10 eV for g-C_3_N_4_ and α-Fe_2_O_3_, respectively.

**Figure 6:**
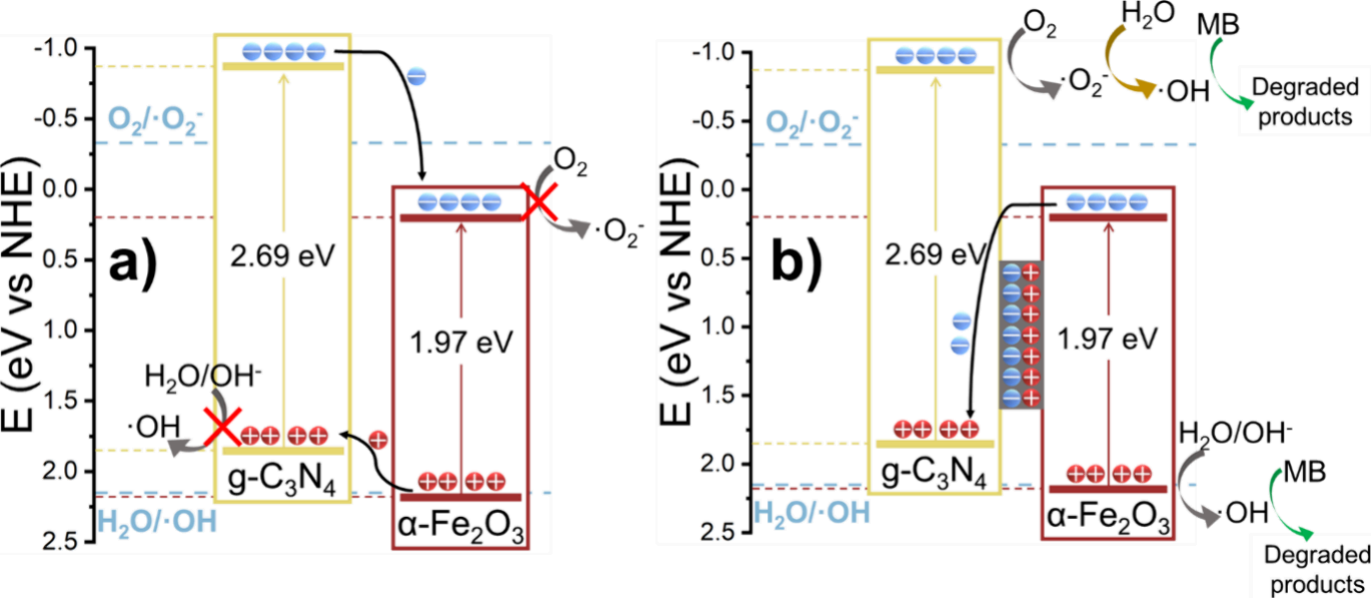
Schematics of two energetically possible heterojunction scenarios for the α-Fe_2_O_3_/g-C_3_N_4_ nanohybrid with respective charge transfer mechanism of photogenerated charges: (a) Type-II heterojunction and (b) Z-scheme heterojunction, indicating adverse and favorable pathways for the generation of reactive oxygen species, respectively.

On the basis of the results presented here, two possible scenarios for the mechanisms governing the charge transfer for the photocatalytic degradation of MB may be considered, namely, a type-II heterojunction or a direct Z-scheme heterojunction. In the first case (Figure 6a), the photogenerated holes in α-Fe_2_O_3_ would migrate to the valence band of g-C_3_N_4_, while the electrons would follow the opposite pathway, from the conduction band of g-C_3_N_4_ to that of α-Fe_2_O_3_. However, this scenario would not allow the formation of reactive oxygen species, as the valence band of g-C_3_N_4_ and the conduction band of α-Fe_2_O_3_ both lie below the energy levels required to convert H_2_O into •OH and O_2_ into •O_2_^−^ radicals, respectively. Since the photocatalytic degradation of MB was proven by ESR to be driven by the formation of these reactive oxygen species, the charge transfer of the nanohybrid photocatalyst must follow a Z-scheme pathway (Figure 6b). In this case, the photogenerated holes of α-Fe_2_O_3_ remain in the valence band while photogenerated electrons transfer from the conduction band to the valence band of g-C_3_N_4_. This results in the formation of an internal electric field at the materials interface, fostered by the transfer of electrons from α-Fe_2_O_3_ to g-C_3_N_4_, that promotes charge transfer and suppresses charge recombination. Therefore, the holes in the valence band of α-Fe_2_O_3_ and the electrons in the conduction band of g-C_3_N_4_ are efficiently separated, preventing recombination. As a result, these separated electrons and holes can now efficiently generate •OH and •O_2_^−^ from H_2_O and O_2_, providing the reactive oxygen species that are needed to drive the photocatalytic degradation of MB.

## 4 Conclusions

This work presents an innovative approach for the growth of α-Fe_2_O_3_/g-C_3_N_4_ nanohybrid photocatalysts with controlled nanoparticle size and phase, yielding an enhanced photocatalytic activity and stability toward the degradation of organic dyes. The origin of this improvement lies in the formation of a direct Z-scheme heterojunction that synergistically enhances the photogeneration, separation, and transfer of charges, afforded by the internal electric field developed at the contact interface between α-Fe_2_O_3_ and g-C_3_N_4_ in the nnaohybrid. This, in turn, promotes the increased formation of radicals required in the photo-Fenton process herein studied for the photocatalytic degradation of pollutants. Thus, the optimum hematite g-C_3_N_4_/α-Fe_2_O_3_ 0.25 M nanohybrid reveals an enhanced photocatalytic degradation activity with respect to reference materials, reducing the overall degradation time from its original 90 min down to 60 min. The Z-scheme heterojunction is formed by a novel two-step methodology that involves an initial ultrasound-induced impregnation of g-C_3_N_4_ employing an aqueous FeCl_3_ precursor solution, followed by a mild microwave-driven phase-conversion treatment. The first step ensures the successful anchoring of Fe-species by the formation of Fe−N and C−N/C−O bonds onto cleaved g-C_3_N_4_ sheets. In the second step, the impregnated Fe species are effectively converted into hematite nanoparticles, whose size is critically controlled by the concentration of the employed FeCl_3_ precursor solution, directly affecting their photocatalytic performance. An upper concentration threshold value of 0.25 M affords a maximum loading of 11 wt % of well-integrated α-Fe_2_O_3_ nanoparticles that maximizes the photocatalytic activity and stability of the resulting nanohybrid. This significant improvement is attributed to the intimate interaction between the hematite nanoparticles and g-C_3_N_4_ sheets. Our two-step approach offers a facile, efficient, and scalable route for the controlled growth of hematite phase-pure g-C_3_N_4_/α-Fe_2_O_3_ nanohybrids, being exploited as highly active Z-scheme photocatalysts in photo-Fenton processes for the sustainable degradation of organic contaminants.

## Supporting Information Available

Detailed structural information obtained from XRD and Rietveld refinement of the hybrids, UV−vis spectra of all the as-prepared photocatalysts, FESEM images of the control and hybrid photocatalysts, schematic of the g-C_3_N_4_ cleavage and impregnation, additional XPS spectra of α-Fe_2_O_3_ NPs and α-Fe_2_O_3_/g-C_3_N_4_ hybrid, additional degradation experiments, and table comparing the photocatalytic activity of different material systems (PDF)

## References

[ref1] Walling C. Fenton’s Reagent Revisited. Acc. Chem. Res. 1975, 8 (4), 125–131. 10.1021/ar50088a003.

[ref2] Chamarro E.; Marco A.; Esplugas S. Use of Fenton Reagent to Improve Organic Chemical Biodegradability. Water Res. 2001, 35 (4), 1047–1051. 10.1016/S0043-1354(00)00342-0. 11235870

[ref3] Liu Y.; Jin W.; Zhao Y.; Zhang G.; Zhang W. Enhanced Catalytic Degradation of Methylene Blue by α-Fe2O3/Graphene Oxide via Heterogeneous Photo-Fenton Reactions. Appl. Catal., B 2017, 206, 642–652. 10.1016/j.apcatb.2017.01.075.

[ref4] Zhang G.; Gao Y.; Zhang Y.; Guo Y. Fe2O3-Pillared Rectorite as an Efficient and Stable Fenton-Like Heterogeneous Catalyst for Photodegradation of Organic Contaminants. Environ. Sci. Technol. 2010, 44 (16), 6384–6389. 10.1021/es1011093. 20704239

[ref5] Xiao C.; Li J.; Zhang G. Synthesis of Stable Burger-like α-Fe2O3 Catalysts: Formation Mechanism and Excellent Photo-Fenton Catalytic Performance. J. Clean. Prod. 2018, 180, 550–559. 10.1016/j.jclepro.2018.01.127.

[ref6] Oller I.; Malato S. Photo-Fenton Applied to the Removal of Pharmaceutical and Other Pollutants of Emerging Concern. Curr. Opin. Green Sustain. Chem. 2021, 29, 100458 10.1016/j.cogsc.2021.100458.

[ref7] Li J.; You J.; Wang Z.; Zhao Y.; Xu J.; Li X.; Zhang H. Application of α-Fe2O3-Based Heterogeneous Photo-Fenton Catalyst in Wastewater Treatment: A Review of Recent Advances. J. Environ. Chem. Eng. 2022, 10 (5), 108329 10.1016/j.jece.2022.108329.

[ref8] Morin F. J. Electrical Properties of α-Fe2O3 and α-Fe2O3 Containing Titanium. Phys. Rev. 1951, 83 (5), 1005–1011. 10.1103/PhysRev.83.1005.

[ref9] Kennedy J. H.; Frese K. W. Photooxidation of Water at α-Fe2O3 Photoanodes. J. Electrochem. Soc. 1978, 125 (5), 709–714. 10.1149/1.2131532.

[ref10] Cherepy N. J.; Liston D. B.; Lovejoy J. A.; Deng H.; Zhang J. Z. Ultrafast Studies of Photoexcited Electron Dynamics in γ- and α-Fe2O3 Semiconductor Nanoparticles. J. Phys. Chem. B 1998, 102 (5), 770–776. 10.1021/jp973149e.

[ref11] Cowan A. J.; Barnett C. J.; Pendlebury S. R.; Barroso M.; Sivula K.; Grätzel M.; Durrant J. R.; Klug D. R. Activation Energies for the Rate-Limiting Step in Water Photooxidation by Nanostructured α-Fe2O3 and TiO2. J. Am. Chem. Soc. 2011, 133 (26), 10134–10140. 10.1021/ja200800t. 21553825

[ref12] AlSalka Y.; Granone L. I.; Ramadan W.; Hakki A.; Dillert R.; Bahnemann D. W. Iron-Based Photocatalytic and Photoelectrocatalytic Nano-Structures: Facts, Perspectives, and Expectations. Appl. Catal., B 2019, 244, 1065–1095. 10.1016/j.apcatb.2018.12.014.

[ref13] Patra A. K.; Kundu S. K.; Bhaumik A.; Kim D. Morphology Evolution of Single-Crystalline Hematite Nanocrystals: Magnetically Recoverable Nanocatalysts for Enhanced Facet-Driven Photoredox Activity. Nanoscale 2016, 8 (1), 365–377. 10.1039/C5NR06509G. 26616162

[ref14] Huang X.; Chen Y.; Walter E.; Zong M.; Wang Y.; Zhang X.; Qafoku O.; Wang Z.; Rosso K. M. Facet-Specific Photocatalytic Degradation of Organics by Heterogeneous Fenton Chemistry on Hematite Nanoparticles. Environ. Sci. Technol. 2019, 53 (17), 10197–10207. 10.1021/acs.est.9b02946. 31397154

[ref15] Liu H.; Tian K.; Ning J.; Zhong Y.; Zhang Z.; Hu Y. One-Step Solvothermal Formation of Pt Nanoparticles Decorated Pt2+-Doped α-Fe2O3 Nanoplates with Enhanced Photocatalytic O2 Evolution. ACS Catal. 2019, 9 (2), 1211–1219. 10.1021/acscatal.8b03819.

[ref16] Keerthana Sp; Yuvakkumar R.; Ravi G.; Kumar P.; Elshikh M. S.; Alkhamis H. H.; Alrefaei A. F.; Velauthapillai D. A Strategy to Enhance the Photocatalytic Efficiency of α-Fe2O3. Chemosphere 2021, 270, 129498 10.1016/j.chemosphere.2020.129498. 33422995

[ref17] Mohamed H. H.; Alomair N. A.; Akhtar S.; Youssef T. E. Eco-Friendly Synthesized α-Fe2O3/TiO2 Heterojunction with Enhanced Visible Light Photocatalytic Activity. J. Photochem. Photobiol. Chem. 2019, 382, 111951 10.1016/j.jphotochem.2019.111951.

[ref18] Norouzi A.; Nezamzadeh-Ejhieh A. α-Fe2O3/Cu2O Heterostructure: Brief Characterization and Kinetic Aspect of Degradation of Methylene Blue. Phys. B Condens. Matter 2020, 599, 412422 10.1016/j.physb.2020.412422.

[ref19] Wang W.; Zhao W.; Zhang H.; Dou X.; Shi H. 2D/2D Step-Scheme α-Fe2O3/Bi2WO6 Photocatalyst with Efficient Charge Transfer for Enhanced Photo-Fenton Catalytic Activity. Chin. J. Catal. 2021, 42 (1), 97–106. 10.1016/S1872-2067(20)63602-6.

[ref20] Moniz S. J. A.; Shevlin S. A.; Martin D. J.; Guo Z.-X.; Tang J. Visible-Light Driven Heterojunction Photocatalysts for Water Splitting − a Critical Review. Energy Environ. Sci. 2015, 8 (3), 731–759. 10.1039/C4EE03271C.

[ref21] Zhou P.; Yu J.; Jaroniec M. All-Solid-State Z-Scheme Photocatalytic Systems. Adv. Mater. 2014, 26 (29), 4920–4935. 10.1002/adma.201400288. 24888530

[ref22] Low J.; Jiang C.; Cheng B.; Wageh S.; Al-Ghamdi A. A.; Yu J. A Review of Direct Z-Scheme Photocatalysts. Small Methods 2017, 1 (5), 1700080 10.1002/smtd.201700080.

[ref23] Xu Q.; Zhang L.; Yu J.; Wageh S.; Al-Ghamdi A. A.; Jaroniec M. Direct Z-Scheme Photocatalysts: Principles, Synthesis, and Applications. Mater. Today 2018, 21 (10), 1042–1063. 10.1016/j.mattod.2018.04.008.

[ref24] Mu Y.-F.; Zhang C.; Zhang M.-R.; Zhang W.; Zhang M.; Lu T.-B. Direct Z-Scheme Heterojunction of Ligand-Free FAPbBr 3 /α-Fe2O3 for Boosting Photocatalysis of CO2 Reduction Coupled with Water Oxidation. ACS Appl. Mater. Interfaces 2021, 13 (19), 22314–22322. 10.1021/acsami.1c01718. 33961390

[ref25] Ma C.; Lee J.; Kim Y.; Cheol Seo W.; Jung H.; Yang W. Rational Design of α-Fe2O3 Nanocubes Supported BiVO4 Z-Scheme Photocatalyst for Photocatalytic Degradation of Antibiotic under Visible Light. J. Colloid Interface Sci. 2021, 581, 514–522. 10.1016/j.jcis.2020.07.127. 32814183

[ref26] Jia Y.; Zhang W.; Yeon Do J.; Kang M.; Liu C. Z-scheme SnFe2O4/α-Fe2O3Micro-Octahedron with Intimated Interface for Photocatalytic CO2 Reduction. Chem. Eng. J. 2020, 402, 126193 10.1016/j.cej.2020.126193.

[ref27] Guo M.; Xing Z.; Zhao T.; Qiu Y.; Tao B.; Li Z.; Zhou W. Hollow Flower-like Polyhedral α-Fe2O3/Defective MoS2/Ag Z-Scheme Heterojunctions with Enhanced Photocatalytic-Fenton Performance via Surface Plasmon Resonance and Photothermal Effects. Appl. Catal., B 2020, 272, 118978 10.1016/j.apcatb.2020.118978.

[ref28] Long L.; Lv G.; Han Q.; Wu X.; Qian Y.; Wang D.; Zhou Y.; Zou Z. Achieving Direct Z-Scheme Charge Transfer through Constructing 2D/2D α-Fe2O3 /CdS Heterostructure for Efficient Photocatalytic CO2 Conversion. J. Phys. Chem. C 2021, 125 (42), 23142–23152. 10.1021/acs.jpcc.1c06259.

[ref29] Xu Q.; Zhu B.; Jiang C.; Cheng B.; Yu J. Constructing 2D/2D Fe2O3/g-C3N4 Direct Z-Scheme Photocatalysts with Enhanced H2 Generation Performance. Sol. RRL 2018, 2 (3), 1800006 10.1002/solr.201800006.

[ref30] Geng Y.; Chen D.; Li N.; Xu Q.; Li H.; He J.; Lu J. Z-Scheme 2D/2D α-Fe2O3/g-C3N4 Heterojunction for Photocatalytic Oxidation of Nitric Oxide. Appl. Catal., B 2021, 280, 119409 10.1016/j.apcatb.2020.119409.

[ref31] Wang X.; Maeda K.; Thomas A.; Takanabe K.; Xin G.; Carlsson J. M.; Domen K.; Antonietti M. A Metal-Free Polymeric Photocatalyst for Hydrogen Production from Water under Visible Light. Nat. Mater. 2009, 8 (1), 76–80. 10.1038/nmat2317. 18997776

[ref32] Ong W.-J.; Tan L.-L.; Ng Y. H.; Yong S.-T.; Chai S.-P. Graphitic Carbon Nitride (g-C3N4)-Based Photocatalysts for Artificial Photosynthesis and Environmental Remediation: Are We a Step Closer To Achieving Sustainability?. Chem. Rev. 2016, 116 (12), 7159–7329. 10.1021/acs.chemrev.6b00075. 27199146

[ref33] Xu B.; Ahmed M. B.; Zhou J. L.; Altaee A.; Xu G.; Wu M. Graphitic Carbon Nitride Based Nanocomposites for the Photocatalysis of Organic Contaminants under Visible Irradiation: Progress, Limitations and Future Directions. Sci. Total Environ. 2018, 633, 546–559. 10.1016/j.scitotenv.2018.03.206. 29579666

[ref34] Hasija V.; Raizada P.; Sudhaik A.; Sharma K.; Kumar A.; Singh P.; Jonnalagadda S. B.; Thakur V. K. Recent Advances in Noble Metal Free Doped Graphitic Carbon Nitride Based Nanohybrids for Photocatalysis of Organic Contaminants in Water: A Review. Appl. Mater. Today 2019, 15, 494–524. 10.1016/j.apmt.2019.04.003.

[ref35] Yang S.; Guo X.; Liu K.; Li Y.; Li T.; Gu X.; Arenal R.; Zheng X.; Li W.; Sun C.; Wang H.; Huang F. Size Effect of CoS2 Cocatalyst on Photocatalytic Hydrogen Evolution Performance of G-C3N4. J. Colloid Interface Sci. 2023, 635, 305–315. 10.1016/j.jcis.2022.12.149. 36587582

[ref36] She X.; Wu J.; Xu H.; Zhong J.; Wang Y.; Song Y.; Nie K.; Liu Y.; Yang Y.; Rodrigues M. F.; Vajtai R.; Lou J.; Du D.; Li H.; Ajayan P. M. High Efficiency Photocatalytic Water Splitting Using 2D α-Fe2O3/g-C3N4 Z-Scheme Catalysts. Adv. Energy Mater. 2017, 7 (17), 1700025 10.1002/aenm.201700025.

[ref37] Jiang Z.; Wan W.; Li H.; Yuan S.; Zhao H.; Wong P. K. A Hierarchical Z-Scheme α-Fe2O3/g-C3N4 Hybrid for Enhanced Photocatalytic CO2 Reduction. Adv. Mater. 2018, 30 (10), 1706108 10.1002/adma.201706108. 29349885

[ref38] Xi J.; Xia H.; Ning X.; Zhang Z.; Liu J.; Mu Z.; Zhang S.; Du P.; Lu X. Carbon-Intercalated 0D/2D Hybrid of Hematite Quantum Dots/Graphitic Carbon Nitride Nanosheets as Superior Catalyst for Advanced Oxidation. Small 2019, 15 (43), 1902744 10.1002/smll.201902744. 31532897

[ref39] Pham V. V.; Truong T. K.; Hai L. V.; La H. P. P.; Nguyen H. T.; Lam V. Q.; Tong H. D.; Nguyen T. Q.; Sabbah A.; Chen K.-H.; You S.-J.; Cao T. M. S-Scheme α-Fe2O3/g-C3N4 Nanocomposites as Heterojunction Photocatalysts for Antibiotic Degradation. ACS Appl. Nano Mater. 2022, 5 (3), 4506–4514. 10.1021/acsanm.2c00741.

[ref40] Guo H.; Chen M.; Zhong Q.; Wang Y.; Ma W.; Ding J. Synthesis of Z-Scheme α-Fe2O3/g-C3N4 Composite with Enhanced Visible-Light Photocatalytic Reduction of CO2 to CH3OH. J. CO2 Util. 2019, 33, 233–241. 10.1016/j.jcou.2019.05.016.

[ref41] Duan B.; Mei L. A Z-Scheme Fe2O3/g-C3N4Heterojunction for Carbon Dioxide to Hydrocarbon Fuel under Visible Illuminance. J. Colloid Interface Sci. 2020, 575, 265–273. 10.1016/j.jcis.2020.04.112. 32375108

[ref42] Zhang J.; Gou S.; Yang Z.; Li C.; Wang W. Photocatalytic Degradation of Sulfamethoxazole over S-Scheme Fe2O3/g-C3N4 Photocatalyst under Visible Light. Water Cycle 2024, 5, 1–8. 10.1016/j.watcyc.2023.11.001.

[ref43] Kong L.; Yan J.; Li P.; Liu S. F. Fe2 O3 /C−C3 N4 -Based Tight Heterojunction for Boosting Visible-Light-Driven Photocatalytic Water Oxidation. ACS Sustain. Chem. Eng. 2018, 6 (8), 10436–10444. 10.1021/acssuschemeng.8b01799.

[ref44] Wang R.; Dai A.; Vijayalakshmi M.; Reddy K. R.; Tang H.; Cheolho B.; Shim J.; Reddy Ch. V. Fabrication of Z-Scheme Fe2O3/g-C3N4 Nanocomposite with Improved Visible Light Induced Photodegradation of Pharmaceutical Pollutants and Photoelectrochemical Water Oxidation. J. Alloys Compd. 2024, 1005, 176086 10.1016/j.jallcom.2024.176086.

[ref45] Hosseini M.; Ghanbari M.; Alzaidy A. H.; Dawi E. A.; Mahdi M. A.; Jasim L. S.; Sobhani A.; Salavati-Niasari M. Synthesis and Characterization of Fe2SiO4/Fe2O3/g-C3N4 Ternary Heterojunction Photocatalyst with Enhanced Photocatalytic Activity under Visible Light. Int. J. Hydrog. Energy 2024, 60, 1370–1382. 10.1016/j.ijhydene.2024.02.253.

[ref46] Priecel P.; Lopez-Sanchez J. A. Advantages and Limitations of Microwave Reactors: From Chemical Synthesis to the Catalytic Valorization of Biobased Chemicals. ACS Sustain. Chem. Eng. 2019, 7 (1), 3–21. 10.1021/acssuschemeng.8b03286.

[ref47] Mishra A.; Mehta A.; Basu S.; Shetti N. P.; Reddy K. R.; Aminabhavi T. M. Graphitic Carbon Nitride (g-C3N4)−Based Metal-Free Photocatalysts for Water Splitting: A Review. Carbon 2019, 149, 693–721. 10.1016/j.carbon.2019.04.104.

[ref48] Zaluzec N. First Light on the Argonne PicoProbe and The X-Ray Perimeter Array Detector (XPAD). Microsc. Microanal. 2021, 27 (S1), 2070–2074. 10.1017/S1431927621007492.

[ref49] Quitério P.; Apolinário A.; Navas D.; Magalhães S.; Alves E.; Mendes A.; Sousa C. T.; Araújo J. P. Photoelectrochemical Water Splitting: Thermal Annealing Challenges on Hematite Nanowires. J. Phys. Chem. C 2020, 124 (24), 12897–12911. 10.1021/acs.jpcc.0c01259.

[ref50] Fina F.; Callear S. K.; Carins G. M.; Irvine J. T. S. Structural Investigation of Graphitic Carbon Nitride via XRD and Neutron Diffraction. Chem. Mater. 2015, 27 (7), 2612–2618. 10.1021/acs.chemmater.5b00411.

[ref51] Ge L. Synthesis and Photocatalytic Performance of Novel Metal-Free g-C3N4 Photocatalysts. Mater. Lett. 2011, 65 (17−18), 2652–2654. 10.1016/j.matlet.2011.05.069.

[ref52] Li Z.; Lin W.; Moon K.-S.; Wilkins S. J.; Yao Y.; Watkins K.; Morato L.; Wong C. Metal Catalyst Residues in Carbon Nanotubes Decrease the Thermal Stability of Carbon Nanotube/Silicone Composites. Carbon 2011, 49 (13), 4138–4148. 10.1016/j.carbon.2011.05.042.

[ref53] Li Y.; He Z.; Liu L.; Jiang Y.; Ong W.-J.; Duan Y.; Ho W.; Dong F. Inside-and-out Modification of Graphitic Carbon Nitride (g-C3N4) Photocatalysts via Defect Engineering for Energy and Environmental Science. Nano Energy 2023, 105, 108032 10.1016/j.nanoen.2022.108032.

[ref54] Jorge A. B.; Martin D. J.; Dhanoa M. T. S.; Rahman A. S.; Makwana N.; Tang J.; Sella A.; Corà F.; Firth S.; Darr J. A.; McMillan P. F. H2 and O2 Evolution from Water Half-Splitting Reactions by Graphitic Carbon Nitride Materials. J. Phys. Chem. C 2013, 117 (14), 7178–7185. 10.1021/jp4009338.

[ref55] Peng B.; Zhang S.; Yang S.; Wang H.; Yu H.; Zhang S.; Peng F. Synthesis and Characterization of G-C3N4/Cu2O Composite Catalyst with Enhanced Photocatalytic Activity under Visible Light Irradiation. Mater. Res. Bull. 2014, 56, 19–24. 10.1016/j.materresbull.2014.04.042.

[ref56] Yang S.; Gong Y.; Zhang J.; Zhan L.; Ma L.; Fang Z.; Vajtai R.; Wang X.; Ajayan P. M. Exfoliated Graphitic Carbon Nitride Nanosheets as Efficient Catalysts for Hydrogen Evolution Under Visible Light. Adv. Mater. 2013, 25 (17), 2452–2456. 10.1002/adma.201204453. 23450777

[ref57] Marshall-Roth T.; Libretto N. J.; Wrobel A. T.; Anderton K. J.; Pegis M. L.; Ricke N. D.; Voorhis T. V.; Miller J. T.; Surendranath Y. A Pyridinic Fe-N4Macrocycle Models the Active Sites in Fe/N-Doped Carbon Electrocatalysts. Nat. Commun. 2020, 11 (1), 5283 10.1038/s41467-020-18969-6. 33077736 PMC7572418

[ref58] Elbanna O.; Fujitsuka M.; Majima T. G-C3N4/TiO2Mesocrystals Composite for H2 Evolution under Visible-Light Irradiation and Its Charge Carrier Dynamics. ACS Appl. Mater. Interfaces 2017, 9 (40), 34844–34854. 10.1021/acsami.7b08548. 28914526

[ref59] Wang W.; Zhang H.; Zhang S.; Liu Y.; Wang G.; Sun C.; Zhao H. Potassium-Ion-Assisted Regeneration of Active Cyano Groups in Carbon Nitride Nanoribbons: Visible-Light-Driven Photocatalytic Nitrogen Reduction. Angew. Chem., Int. Ed. 2019, 58 (46), 16644–16650. 10.1002/anie.201908640. 31497911

[ref60] Xu H.; Zhang T.; Gu Y.; Yan X.; Lu N.; Liu H.; Xu Z.; Xing Y.; Song Y.; Zhang Z.; Yang M. An Electrochemical Thrombin Aptasensor Based on the Use of Graphite-like C3N4Modified with Silver Nanoparticles. Microchim. Acta 2020, 187 (3), 163 10.1007/s00604-020-4111-4. 32052190

[ref61] Gao J.; Wang Y.; Zhou S.; Lin W.; Kong Y. A Facile One-Step Synthesis of Fe-Doped g-C3N4 Nanosheets and Their Improved Visible-Light Photocatalytic Performance. ChemCatChem. 2017, 9 (9), 1708–1715. 10.1002/cctc.201700492.

[ref62] Qiu P.; Chen H.; Xu C.; Zhou N.; Jiang F.; Wang X.; Fu Y. Fabrication of an Exfoliated Graphitic Carbon Nitride as a Highly Active Visible Light Photocatalyst. J. Mater. Chem. A 2015, 3 (48), 24237–24244. 10.1039/C5TA08406G.

[ref63] Ouyang J.; Zhao Z.; Suib S. L.; Yang H. Degradation of Congo Red Dye by a Fe2O3@CeO2-ZrO2/Palygorskite Composite Catalyst: Synergetic Effects of Fe2O3. J. Colloid Interface Sci. 2019, 539, 135–145. 10.1016/j.jcis.2018.12.052. 30579217

[ref64] Makino K.; Hagiwara T.; Murakami A. A Mini Review: Fundamental Aspects of Spin Trapping with DMPO. Int. J. Radiat. Appl. Instrum. Part C Radiat. Phys. Chem. 1991, 37 (5−6), 657–665. 10.1016/1359-0197(91)90164-W.

[ref65] Villamena F. A. Reactive Species Detection in Biology: From Fluorescence to Electron Paramagnetic Resonance Spectroscopy; Elsevier: Amsterdam, 2017.

[ref66] Clément J.-L.; Ferré N.; Siri D.; Karoui H.; Rockenbauer A.; Tordo P. Assignment of the EPR Spectrum of 5,5-Dimethyl-1-Pyrroline N -Oxide (DMPO) Superoxide Spin Adduct. J. Org. Chem. 2005, 70 (4), 1198–1203. 10.1021/jo048518z. 15704951

[ref67] Xu C.; Chen Y.; Xie X.; Yan K.; Si Y.; Zhang M.; Yan Q. Construction of Ag SPR-Promoted Z-scheme Ag2MoO4/CuBi2O4 Composites with Enhanced Photocatalytic Performance. J. Mater. Sci. Mater. Electron. 2020, 31 (11), 8151–8164. 10.1007/s10854-020-03271-4.

[ref68] Zhou S.; Wang Y.; Zhou K.; Ba D.; Ao Y.; Wang P. In-Situ Construction of Z-Scheme g-C3N4/WO3 Composite with Enhanced Visible-Light Responsive Performance for Nitenpyram Degradation. Chin. Chem. Lett. 2021, 32 (7), 2179–2182. 10.1016/j.cclet.2020.12.002.

