# Supplementary material for: Fabrication of α-Fe2O3 Nanoparticles/g-C3N4 Direct Z-Scheme Heterojunction of Durable Photocatalytic Activity
Source: ACS Appl Nano Mater. 2025 Apr 29;8(18):9364–75. doi: 10.1021/acsanm.5c00991 (PMC12070367; doi:10.1021/acsanm.5c00991)
Supplement: Supplementary file 1 — an5c00991_si_001.pdf [file an5c00991_si_001.pdf]

SUPPORTING INFORMATION

Fabrication of α-Fe_2O_3 Nanoparticles/g-C_3N_4 Direct Z-Scheme Heterojunction of Durable Photocatalytic Activity

Alejandro Galán-González, [*,†] Isaías Fernández, [†] Nestor J. Zaluzec, [‡] Sofie Cambré, [$] Raul Arenal, [∥,⊥ #] Ana M. Benito, [*,†] Wolfgang K. Maser [*,†]

[†] Instituto de Carboquímica (ICB-CSIC), C/ Miguel Luesma Castán 4, 50018 Zaragoza, Spain

[‡] University of Chicago, Pritzker School of Molecular Engineering and Argonne National Laboratory / Photon Science Directorate, Lemont, Illinois, USA

[$] Theory and Spectroscopy of Molecules and Materials, Department of Physics, University of Antwerp, 2610 Antwerp, Belgium

[∥] Instituto de Nanociencia y Materiales de Aragón (INMA), CSIC-Universidad de Zaragoza, C/ Pedro Cerbuna 12, 50009 Zaragoza, Spain

[⊥] Laboratorio de Microscopias Avanzadas (LMA), Universidad de Zaragoza, C/ Mariano Esquillor s/n, 50018 Zaragoza, Spain

[#] ARAID Foundation, 50018 Zaragoza, Spain

*Corresponding Authors: agalan@icb.csic.es; abenito@icb.csic.es; wmaser@icb.csic.es

Table of contents

S1. X-ray diffraction

Figure S1. XRD pattern of the (1 0 0) peak of g-C_3N_4 and hybrid materials.

Table S1. Crystallographic parameters of hematite NPs and hybrid materials.

S2. UV-vis absorption spectroscopy

Figure S2. UV-vis spectra of the reference and hybrid materials.

S3. XPS of Fe 2p core spectra

Figure S3. Fe 2p XPS spectra of α-Fe_2O_3 NPs and α-Fe_2O_3/g-C_3N_4 hybrid.

S4. Cleavage and impregnation of g-C_3N_4

Figure S4. Schematic of the cleavage and impregnation process of the g-C_3N_4 sheets**.**

S5. High resolution FESEM

Figure S5. High-resolution FESEM micrographs of α-Fe_2O_3 NPs, g-C_3N_4 and hybrids.

S6. Additional photocatalytic MB degradation results

Figure S6. Effect of H_2O_2 and UV light on MB degradation.

Figure S7. Comparison between UV and white ligh on MB degradation.

Figure S8. MB degradation using different concentrations of photocatalyst.

Figure S9. Comparative degradation of MB, RhB and MR.

Figure S10. XRD analysis of the photocatalyst after recycling experiment.

Figure S11. UV-vis spectra of time-dependent MB degradation.

S7. Comparative table

Table S2. Comparison of the photocatalytic activity and degradation time employing different material systems and experimental conditions.

S8. References

S1. X-ray diffraction

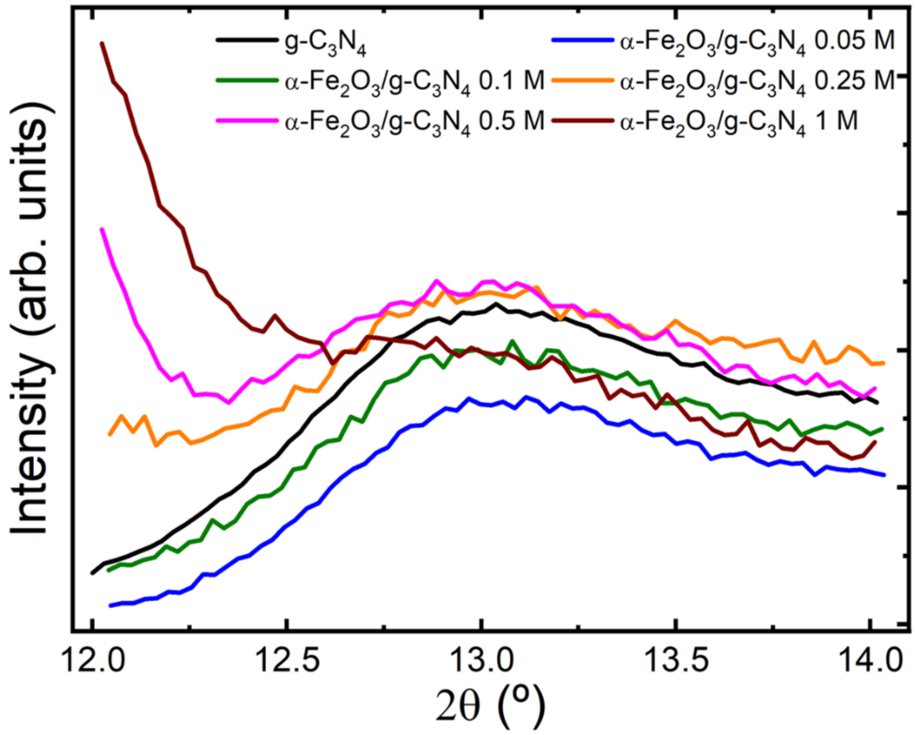

Figure S1. XRD pattern of the (1 0 0) peak for g-C_3N_4 and the α-Fe_2O_3/g-C_3N_4 hybrids as a function of the $FeCl_3$ precursor concentration. The broadening of the peak width accompanied by a lower intensity with increasing Fe concentration indicates a more defective structure i.e., a smaller size of the intraplanar conjugated system.

Crystallographic parameters and XRD of the (1 0 0) peak

Table S1. Summary of the crystallographic parameters (lattice parameters, crystalline domain size and α-Fe_2O_3 content) of the reference and hybrid materials.

Sample	a (Å)	c (Å)	Crystalline domain size (nm)	α-Fe_2O_3	β-FeOOH
β-FeOOH	5.034	13.77	23.7	36.1 %	63.9 %
α-Fe_2O_3	5.035	13.75	115.8	100 %	-
α-Fe_2O_3/g-C_3N_4 0.05 M	5.035	13.75	39.8	100 %	-
α-Fe_2O_3/g-C_3N_4 0.1 M	5.036	13.76	55.4	100 %	-
α-Fe_2O_3/g-C_3N_4 0.25 M	5.039	13.77	66.2	98%	2%
α-Fe_2O_3/g-C_3N_4 0.5 M	5.037	13.77	53.0	55.3 %	44.7 %
α-Fe_2O_3/g-C_3N_4 1 M	5.035	13.75	53.4	65.7 %	34.3 %

S2. UV-vis absorption spectroscopy

The UV-Vis absorption spectra of the control materials are shown in Fig. S3a. The spectrum of the α-Fe_2O_3 nanoparticles shows two shoulders at 510 nm and 410 nm that correspond with the indirect Fe^{3+} d→d and the direct O^{2-} p→ Fe^{3+} d transitions, respectively.[1,2] Regarding the spectrum of g-C_3N_4, two clear signals corresponding with to π→ π* and the n→ π* transitions, located at 360 nm and 500 nm, respectively, are observed.[3] The former is associated with the conjugated heterocyclic ring systems while the latter is a transition originated by lone pairs in solitary N atoms (amino groups) on the edge of the heptazine rings of carbon nitride. The presence of this n→ π* transition is an indication of a distorted heptazine structure that reveals the presence of individual N atoms on the edges of the stacked sheets, which is a deviation from the perfect heptazine structure.[4]

Assessing the UV-Vis absorption of the nanohybrids (Fig. S3b), there is a clear distinction dependent on the presence of iron oxide phase. On the hybrids with pure α-Fe_2O_3 phase, only the π→ π* transition ascribed to g-C_3N_4 can be seen in all nanohybrids, indicating that the defects in the structure of g-C_3N_4 have probably been used as anchoring sites upon which the α-Fe_2O_3 nanoparticles are grown. However, the nanohybrids that contain a mixed α-Fe_2O_3/β-FeOOH phase showed a very different behaviour, highlighted in particular by the appearance of the prominent n→ π* transition. The presence of this transition and the defective g-C_3N_4 correlates well with the XRD results of these nanohybrids (Fig. 2b), revealing the disappearance of the (1 0 0) peak of carbon nitride.

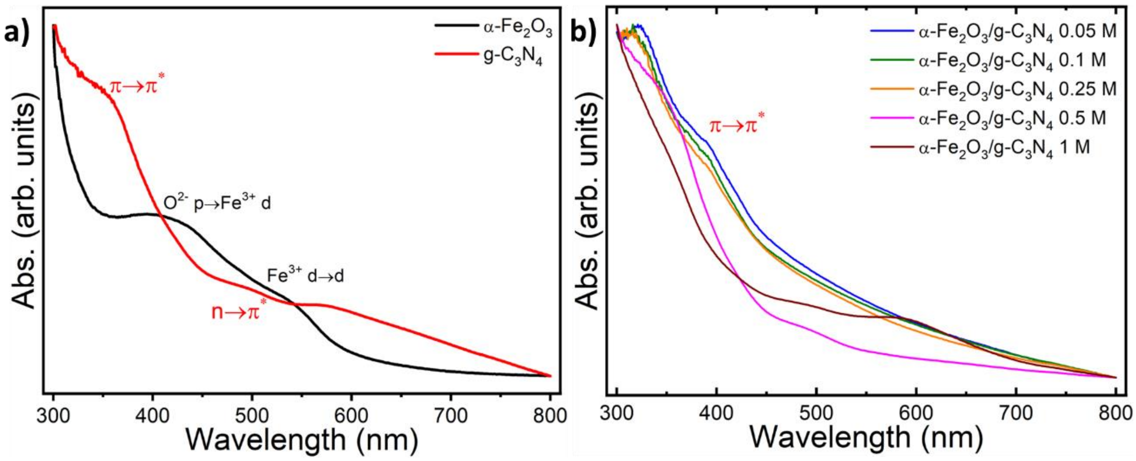

Figure S2. UV-vis absorption spectra of a) g-C_3N_4 and α-Fe_2O_3 nanoparticles, and b) the α-Fe_2O_3/g-C_3N_4 nanohybrid photocatalysts, indicating the assignment of the different transitions.

S3. XPS of Fe 2p core spectra

The Fe 2p peak of the α-Fe_2O_3 NPs (Fig. S5a) and the α-Fe_2O_3/g-C_3N_4 0.25 M hybrid (Fig. S5b) provided further information on any possible change on the iron composition. In both cases, the peak was deconvoluted into six components, with two of them corresponding to the clear satellite signals. Notably, the contribution of the satellite signals diminishes for the hybrid in comparison with the α-Fe_2O_3 nanoparticles.[5] The observed change of the chemical environment for the α-Fe_2O_3 nanoparticles suggest their effective integration with g-C_3N_4, in-line with the alterations in C 1s, N 1s and O1s spectra discussed in the main part of the article.

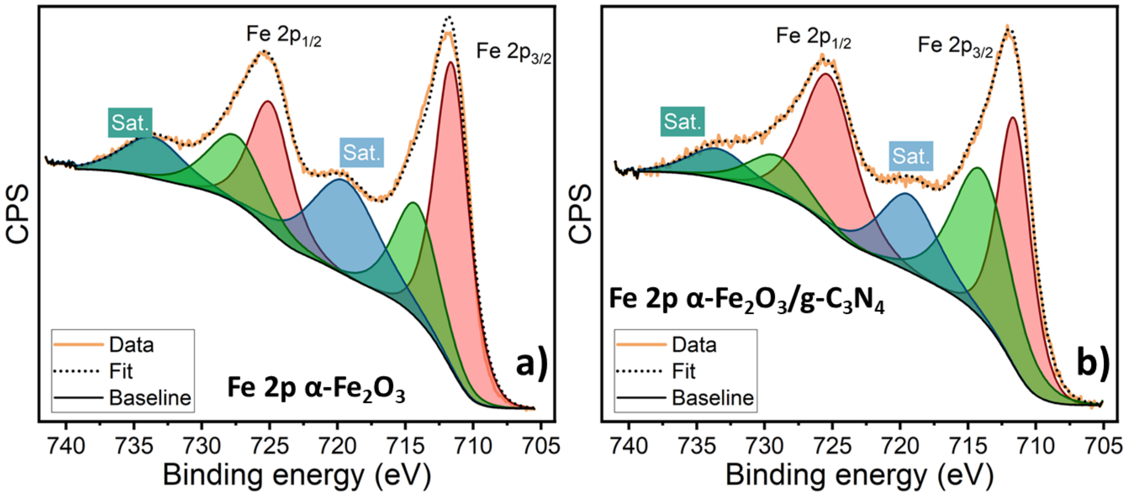

Figure S3. XPS spectra and components of the Fe 2p peak of **(a)** α-Fe_2O_3 NPs and **(b)** α-Fe_2O_3/g-C_3N_4 0.25 M hybrid.

S4. Cleavage and impregnation process of the g-C_3N_4 sheets

The formation mechanism of the α-Fe_2O_3/g-C_3N_4 nanohybrid is depicted in Scheme 1. Here, the double role of the ultrasound-assisted impregnation step is highlighted since it simultaneously induces both the cleavage of the g-C_3N_4 sheets and the formation of very stable Fe-N and C-O/C-N bonds. As a result, the number of catalytically available sites is enhanced while prompting the formation of iron oxide anchoring points on g-C_3N_4.

The subsequent mild microwave treatment converts the anchored Fe-species into α-Fe_2O_3 nanoparticles that are well-integrated onto the g-C_3N_4 sheets, as indicated in Scheme 1 in the main text of the manuscript. The size and phase of these nanoparticles can be directly modulated by controlling the $FeCl_3$ concentration during the impregnation as well as with the duration of the microwave treatment.

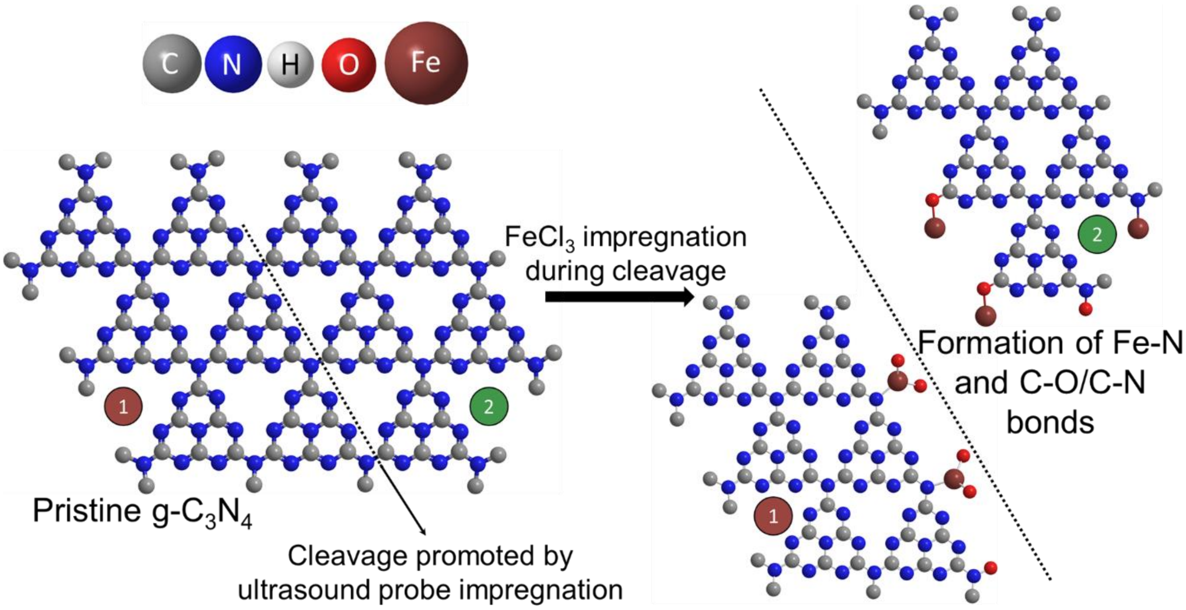

Figure S4. Schematic representation of the g-C_3N_4 cleavage and impregnation process during the ultrasound treatment.

S5. High-resolution FESEM

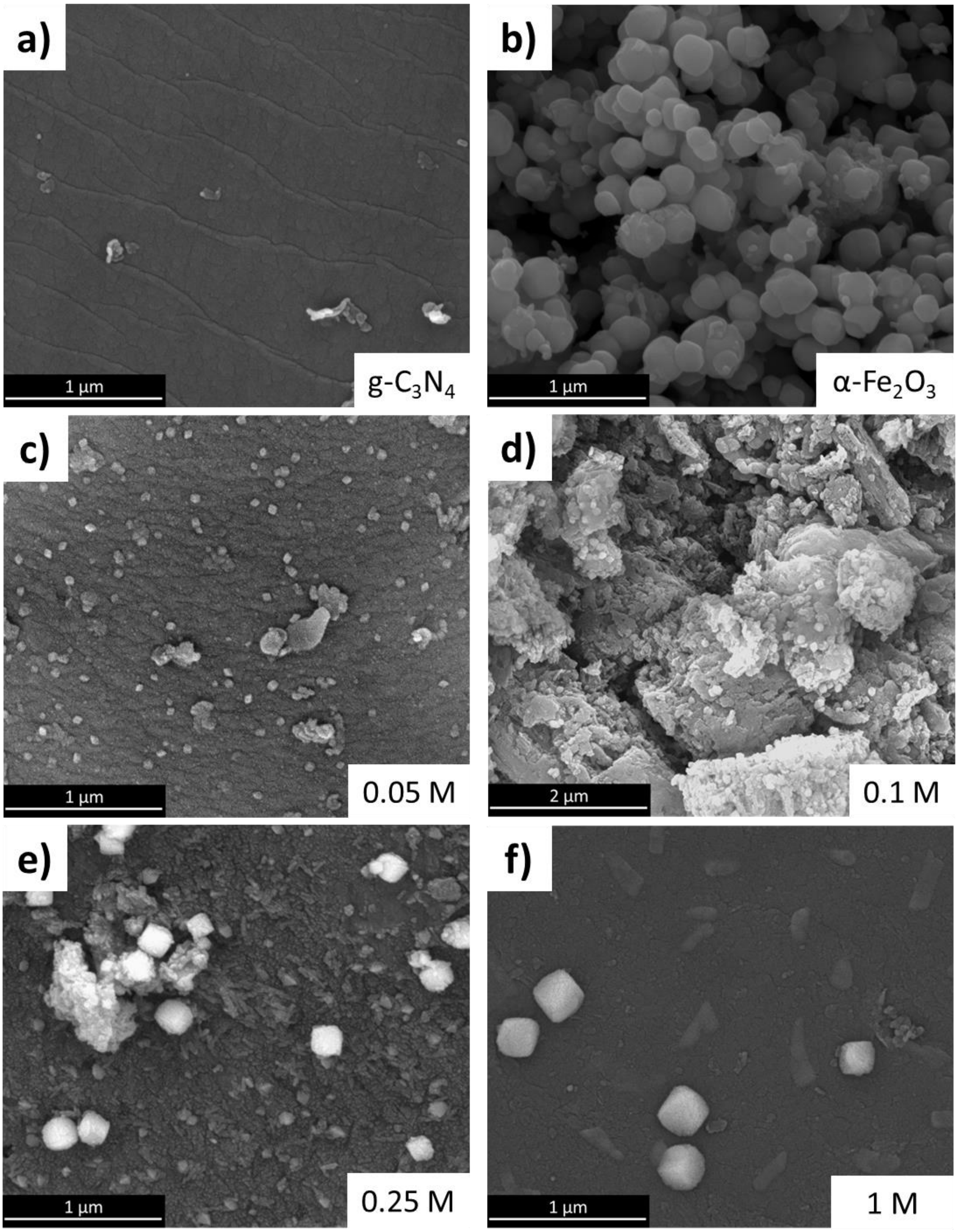

Figure S5. High resolution field emission scanning electron micrographs of (a) α-Fe_2O_3 nanoparticles, (b) surface of a g-C_3N_4 sheet and α-Fe_2O_3/g-C_3N_4 nanohybrid photocatalysts prepared with different concentrations of $FeCl_3$ (c) 0.05 M, (d) 0.1 M, (e) 0.25 M, and (f) 1 M. Tuning the concentration of $FeCl_3$ during the ultrasound probe impregnation clearly provides control over the size of the α-Fe_2O_3 nanoparticles grown onto the surface of g-C_3N_4, which is corroborated by the results detailed in Figure 3 in the main manuscript.

S6. Additional photocatalytic MB degradation results

The MB degradation in absence of either H_2O_2 or UV light was studied for α-Fe_2O_3, g-C_3N_4 and the 0.25 M nanohybrid. α-Fe_2O_3 nanoparticles demonstrate negligible MB degradation in the absence of both UV light or co-oxidant. Conversely, g-C_3N_4 exhibits inherent photocatalytic activity facilitating a significant degradation of MB (40% reduction after 90 minutes) without H_2O_2. However, the addition of H_2O_2 alone, without UV illumination yields negligible degradation over the entire 90-minute period. The α-Fe_2O_3/g-C_3N_4 0.25 M hybrid photocatalyst exhibits an intermediate behavior. A slight degradation of MB is observed in the absence of co-oxidant, while H_2O_2 alone does not lead to a significant dye degradation. These findings highlight the need of having both components simultaneously to achieve an efficient and quick dye degradation.

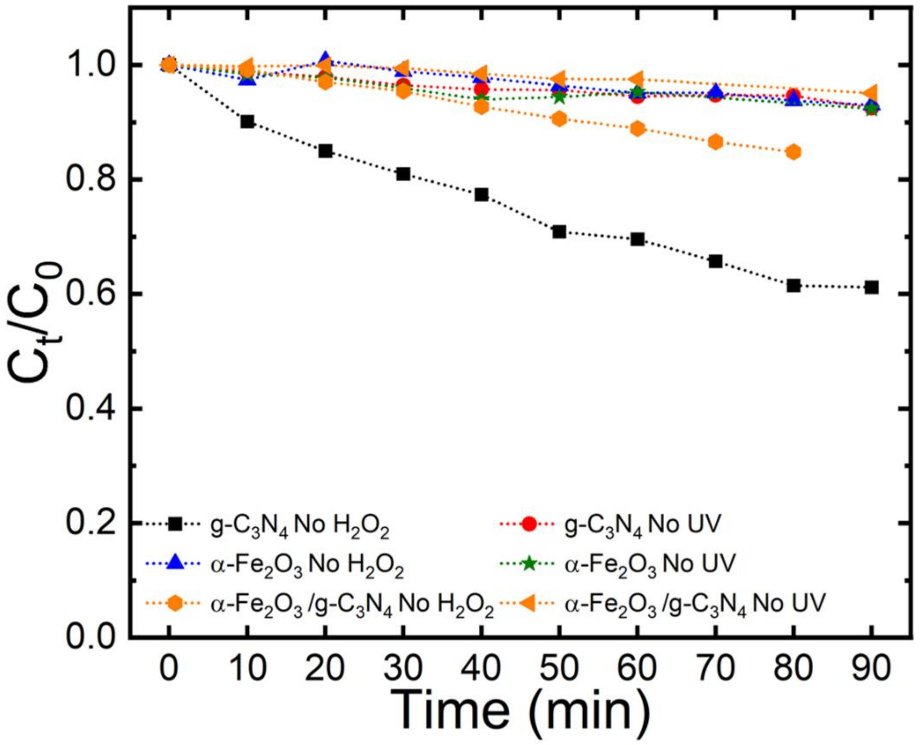

Figure S6. Study of the degradation of MB in absence of either UV light or H_2O_2 as co-oxidant.

Figure S7 shows the degradation of MB while employing either UV light (in black) or white light (in red). As it can be observed, the degradation rate is much faster under UV illumination, ascribed to the enhanced photogeneration of charge carriers prompted by the presence of H_2O_2 and α-Fe_2O_3 via the photo-Fenton process. Under white light illumination, this photo-Fenton effect does not take place and, as such, the degradation rate is significantly reduced. Since the illumination power density for both employed lamps was the same, degradation based on a photolysis effect due to the power density of the illumination can be discarded.

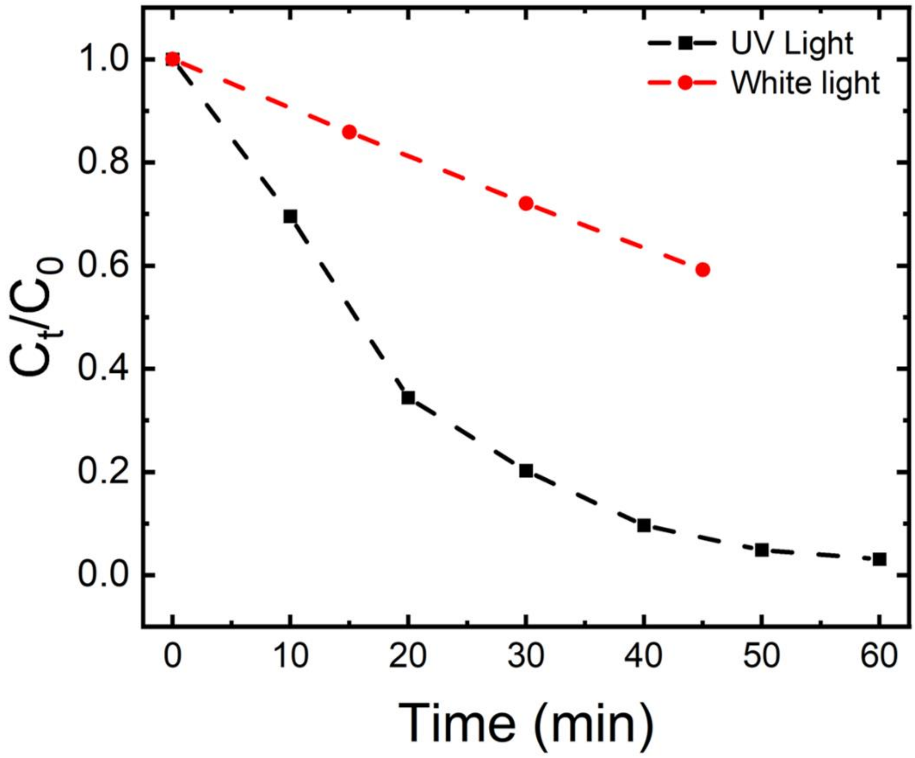

Figure S7. Degradation of MB with the α-Fe_2O_3/g-C_3N_4 0.25 M hybrid using different illumination sources, either UV or white light.

Figure S8 shows the degradation of MB while employing decreasing amounts of the α-Fe_2O_3/g-C_3N_4 0.25 M nanohybrid photocatalyst, from the original 0.5 mg mL^{-1} down to 0.33 mg mL^{-1} d and 0.17 mg mL^{-1}. As expected, the degradation rate decreased with decreasing concentration of the photocatalyst. Nonetheless, the degradation rate with a 0.33 mg mL^{-1} concentration is still faster than any of the other photocatalysts studied. This highlights the outstanding performance of the α-Fe_2O_3/g-C_3N_4 0.25 M nanohybrid since, even after reducing its concentration by one third, it degrades the contaminant at a faster pace than any of the other photocatalysts studied.

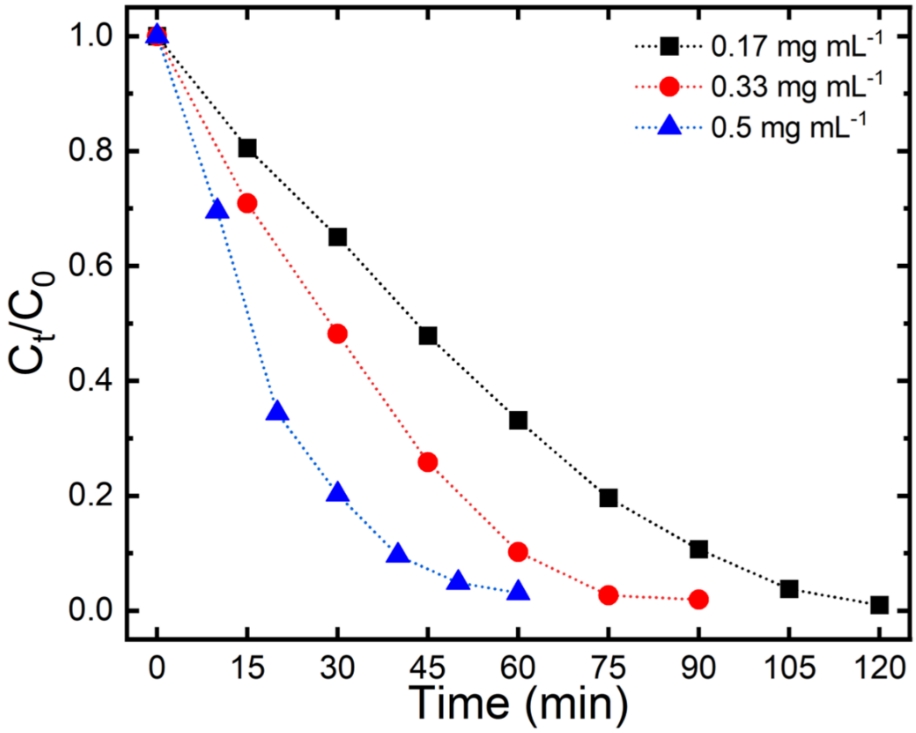

Figure S8. Degradation of MB with the α-Fe_2O_3/g-C_3N_4 0.25 M hybrid using different concentrations of the photocatalyst.

Figure S9 shows the degradation of methylene blue, rhodamine B (RhB) and methyl red (MR) employing the α-Fe_2O_3/g-C_3N_4 0.25 M nanohybrid photocatalyst. Asit can be observed, the two other dyes, RhB and MR, are degraded significantly. The former is completely eliminated, reaching a value very similar to that of MB (close to 100% removal), while MR shows a slower degradation, leaving 35% of the initial concentration. This result highlights the applicability of the as-prepared nanohybrid photocatalyst in the elimination of a variety of organic pollutants.

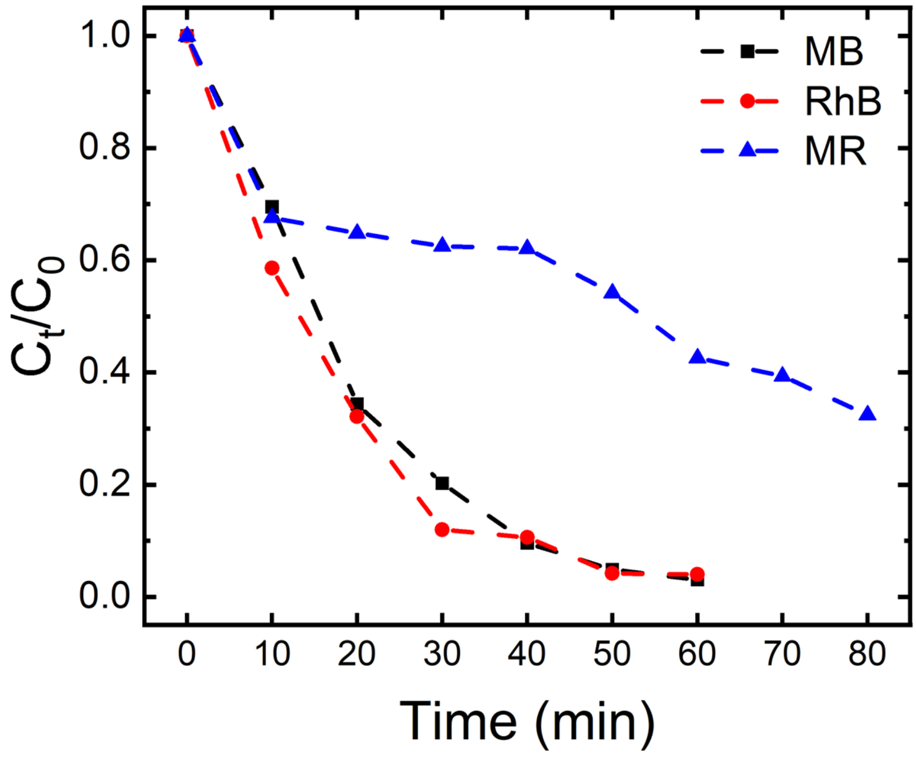

Figure S9. Degradation of MB, RhB and MR with the α-Fe_2O_3/g-C_3N_4 0.25 M nanohybrid photocatalyst.

The XRD pattern of the α-Fe_2O_3/g-C_3N_4 0.25 M photocatalyst before and after the recycling experiment shows minimal differences (Fig. S10). The predominance of g-C_3N_4 is maintained and the hematite signals still represent the majority of the other components. In addition, the small peaks that point towards the presence of residual akageneite can still be seen. All in all, the nanohybrid photocatalyst is completely stable not only from the catalytic point of view, but also from a structural point of view.

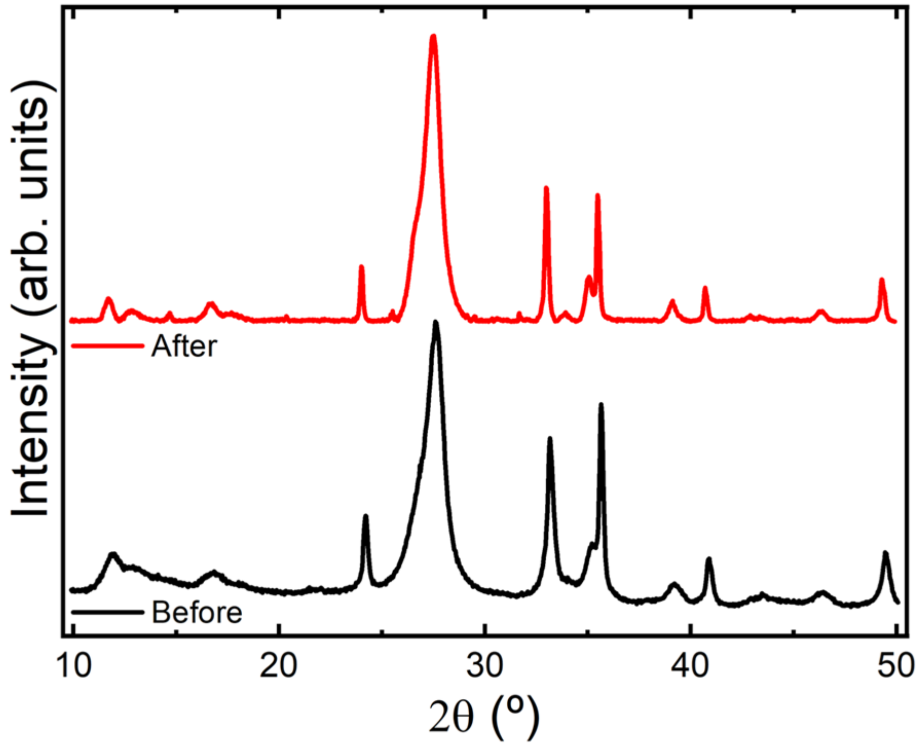

Figure S10. XRD analysis of the α-Fe_2O_3/g-C_3N_4 0.25 M nanohybrid before and after the recycling experiment shown in Fig. 4c.

Example of a typical MB degradation experiment followed by UV-vis spectroscopy.

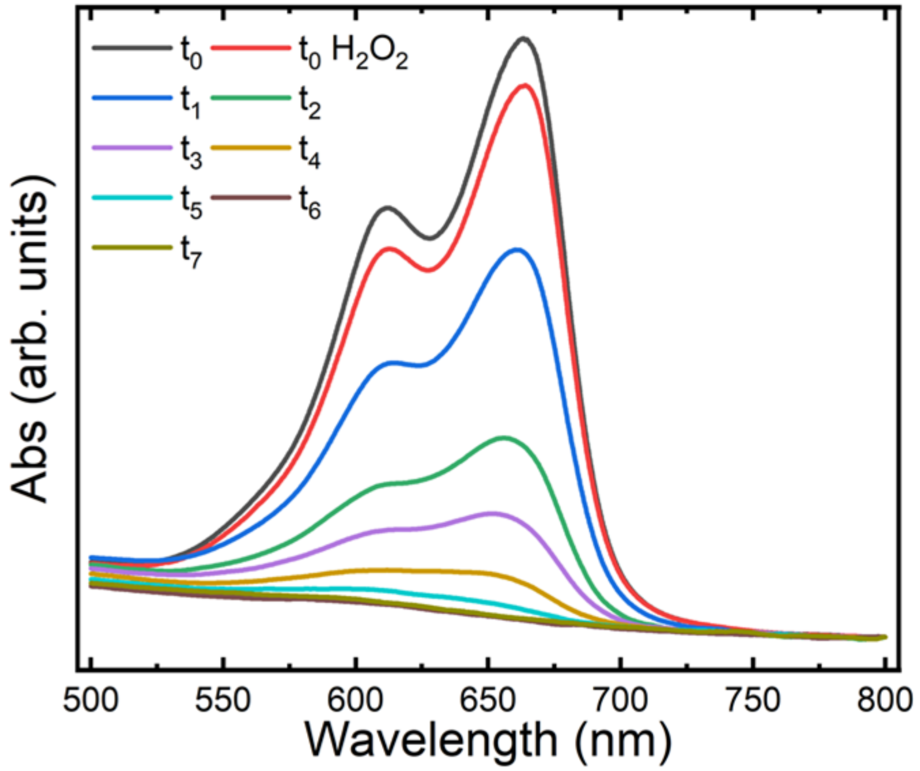

Figure S11. UV-vis spectroscopy curves showing the degradation over time of MB. In this figure, t_i indicates intervals of 15 minutes.

S7. Comparative table

Table S2 shows a comparison of a variety of material systems and heterojunctions (mostly g-C_3N_4-Fe_2O_3 or related materials) and their applicability in the photodegradation of organic pollutants (dyes in most of the examples here included). The preparation method of each photocatalyst is presented, whereby it can be seen that a majority of the works require the use of high-temperature conventional thermal treatments to obtain a successful photocatalytic system. In comparison with the works prsented here, our α-Fe_2O_3/g-C_3N_4 nanohybrid photocatalyst yields an extremely fast and complete photodegradation of the dye using low photocatalyst concentration and a mild power density of the illumination, especially in comparison with the systems that use white light ($\lambda > 400$ nm).

Table S2. Comparison of the photocatalytic activity and degradation time employing different material systems, prepartion pathways and experimental conditions (pollutnat/photocatalyst concentration, illumination).

Photocatalyst	Preparation	Pollutant (concentration)	Catalyst (g L^{-1})	Light source	Time (min)	Removal (%)	Reference
$Fe_2O_{3-x}S_x$/S@g-C_3N_4	Mixture and thermal treatment of precursors	MB (8 mg L^{-1})	0.7	300 W halogen lamp ($\lambda > 400$ nm)	150	82	6
α-Fe_2O_3/CdS/g-C_3N_4	Ultrasonic mixing of as-prepared materials	MB (10 mg L^{-1})	1	1000 W xenon lamp ($\lambda > 300$ nm)	120	100	7
α-Fe_2O_3-g-C_3N_4 (FOCN)	Mixture g-C_3N_4 and α-Fe_2O_3 powders	Direct red 81 (50 mg L^{-1})	1	100 W tungsten - halogen lamp ($\lambda > 400$ nm)	120	75	8
Fe(0.5%)–CN	Thermal treatment of $Fe(NO_3)_3 \cdot 9H_2O$ impregnated melamine	RhB (10 mg L^{-1})	0.25	250 W high-pressure sodium lamp ($\lambda > 400$ nm)	120	100	9
5 wt% FCG (α-Fe_2O_3-CdS-g-C_3N_4)	Solid state mixing and high temperature thermal treatment	MB/RhB (10 mg L^{-1})	1	500 W tungsten - halogen lamp ($\lambda > 400$ nm)	50/25	100	10

Fe_2O_3/g-C_3N_4 (1.0%)	Microwave assisted integration of α-Fe_2O_3 and g-C_3N_4 dispersed powders	MB (30 mg L^{-1})	1	50 W LED lamp (λ > 400 nm)	90	70	11
Fe_2O_3/EuVO4/g-C_3N_4	Sonochemical assisted	RhB (5 mg L^{-1})	0.5	400 W mercury lamp (λ > 400 nm)	120	77	12
α-Fe_2O_3-g-C_3N_4	Mixture g-C_3N_4 and α-Fe_2O_3 powders and thermal treatment	Diazinon (100 mg L^{-1})	0.4	UV Lamp (λ > 300 nm)	60	100	13
Bi@CNT-α-Fe_2O_3	Sonication mixture and laser irradiation	MB (20 mg L^{-1})	0.2	8 W UV lamp (λ = 365 nm)	20	100	14
Fe_3O_4/GO	Sonication and hydrothermal synthesis	MB (40 mg L^{-1})	2.5	100 W mercury lamp (λ = 365 nm)	80	100	15
Fe_3O_4–GO-C_3N_4	Microwave assisted synthesis followed by liquid-phase decoration	MB (10 mg L^{-1})	1	Six 15 W UV lamps (λ = 365 nm)	30	100	16
α-Fe_2O_3-g-C_3N_4	Hydrothermal reaction and calcination	Phenol (50 mg L^{-1})	0.1	350 W Xenon lamp	70	90	17
**α-Fe_2O_3/g-C_3N_4 nanohybrid**	**Ultrasound impregnation and short microwave treatment**	**MB/RhB (15 mg L^{-1})**	**0.5**	**8 W UV lamp (λ = 365 nm)**	**60**	**100%**	**This work**

S8. REFERENCES

(1) Sherman, D. M. The Electronic Structures of Fe^{3+} Coordination Sites in Iron Oxides: Applications to Spectra, Bonding, and Magnetism. *Phys. Chem. Miner.* **1985**, *12* (3), 161–175.

(2) Hahn, N. T.; Ye, H.; Flaherty, D. W.; Bard, A. J.; Mullins, C. B. Reactive Ballistic Deposition of α-Fe_2O_3 Thin Films for Photoelectrochemical Water Oxidation. *ACS Nano* **2010**, *4* (4), 1977–1986.

(3) Jorge, A. B.; Martin, D. J.; Dhanoa, M. T. S.; Rahman, A. S.; Makwana, N.; Tang, J.; Sella, A.; Corà, F.; Firth, S.; Darr, J. A.; McMillan, P. F. H_2 and O_2 Evolution from Water Half-Splitting Reactions by Graphitic Carbon Nitride Materials. *J. Phys. Chem. C* **2013**, *117* (14), 7178–7185.

(4) Chen, Y.; Wang, B.; Lin, S.; Zhang, Y.; Wang, X. Activation of n → Π* Transitions in Two-Dimensional Conjugated Polymers for Visible Light Photocatalysis. *J. Phys. Chem. C* **2014**, *118* (51), 29981–29989.

(5) Xu, W.; Xue, W.; Huang, H.; Wang, J.; Zhong, C.; Mei, D. Morphology Controlled Synthesis of α-Fe_2O_{3-x} with Benzimidazole-Modified Fe-MOFs for Enhanced Photo-Fenton-like Catalysis. *Appl. Catal. B Environ.* **2021**, *291*, 120129.

(6) Jourshabani, M.; Shariatinia, Z.; Badiei, A. High Efficiency Visible-Light-Driven Fe2O3-xS /S-Doped g-C3N4 Heterojunction Photocatalysts: Direct Z-Scheme Mechanism. *J. Mater. Sci. Technol.* **2018**, *34* (9), 1511–1525.

(7) Yavuz, C.; Erten-Ela, S. Solar Light-Responsive α-Fe2O3/CdS/g-C3N4 Ternary Photocatalyst for Photocatalytic Hydrogen Production and Photodegradation of Methylene Blue. *J. Alloys Compd.* **2022**, *908*, 164584.

(8) Theerthagiri, J.; Senthil, R. A.; Priya, A.; Madhavan, J.; Michael, R. J. V.; Ashokkumar, M. Photocatalytic and Photoelectrochemical Studies of Visible-Light Active α-Fe_2O_3–g-C_3N_4 Nanocomposites. *RSC Adv* **2014**, *4* (72), 38222–38229.

(9) Hu, S.; Jin, R.; Lu, G.; Liu, D.; Gui, J. The Properties and Photocatalytic Performance Comparison of Fe3+-Doped g-C3N4 and Fe2O3/g-C3N4 Composite Catalysts. *RSC Adv.* **2014**, *4* (47), 24863.

(10) Athar, M. S.; Danish, M.; Muneer, M. Fabrication of Visible Light-Responsive Dual Z-Scheme (α-Fe2O3/CdS/g-C3N4) Ternary Nanocomposites for Enhanced Photocatalytic Performance and Adsorption Study in Aqueous Suspension. *J. Environ. Chem. Eng.* **2021**, *9* (4), 105754.

(11) Karimi, M. A.; Iliyat, M.; Atashkadi, M.; Ranjbar, M.; Habibi-Yangjeh, A. Microwave-assisted Synthesis of the FE_2O_3 / G-C_3N_4 Nanocomposites with Enhanced Photocatalytic Activity for Degradation of Methylene Blue. *J. Chin. Chem. Soc.* **2020**, *67* (11), 2032–2041.

(12) Monsef, R.; Ghiyasiyan-Arani, M.; Salavati-Niasari, M. Design of Magnetically Recyclable Ternary Fe_2O_3/$EuVO_4$/g-C_3N_4 Nanocomposites for Photocatalytic and Electrochemical Hydrogen Storage. *ACS Appl. Energy Mater.* **2021**, *4* (1), 680–695.

(13) Al-Musawi, T. J.; Asgariyan, R.; Yilmaz, M.; Mengelizadeh, N.; Asghari, A.; Balarak, D.; Darvishmotevall, M. Synthesis of a Doped α-Fe2O3/g-C3N4 Catalyst for High-Efficiency Degradation of Diazinon Contaminant from Liquid Wastes. *Magnetochemistry* **2022**, *8* (11), 137.

(14) Manda, A. A.; Elsayed, K. A.; Gaya, U. I.; Haladu, S. A.; Ercan, İ.; Ercan, F.; Alheshibri, M.; Al Baroot, A.; Kayed, T. S.; Alshammery, S.; Altamimi, N. A.; Al-Otaibi, A. L. Enhanced Photocatalytic Degradation of Methylene Blue by Nanocomposites Prepared by Laser Ablation of Bi on CNT-α-Fe2O3 Nanoparticles. *Opt. Laser Technol.* **2022**, *155*, 108430.

(15) Liu, Y.; Jin, W.; Zhao, Y.; Zhang, G.; Zhang, W. Enhanced Catalytic Degradation of Methylene Blue by α-Fe_2O_3/Graphene Oxide via Heterogeneous Photo-Fenton Reactions. *Appl. Catal. B Environ.* **2017**, *206*, 642–652.
(16) Silva, J. M. P.; Neto, N. F. A.; Lima, A. B.; Correa, M.; Bomio, M. R. D.; Motta, F. V. Investigating Adsorption/Photocatalysis of Organic Contaminants by Fe3O4–GO, Fe3O4–C3N4, and Fe3O4–GO-C3N4 Heterojunctions. *Chem. Inorg. Mater.* **2023**, *1*, 100014.
(17) Ge, F.; Li, X.; Wu, M.; Ding, H.; Li, X. A Type II Heterojunction α-Fe_2O_3/g-C_3N_4 for the Heterogeneous Photo-Fenton Degradation of Phenol. *RSC Adv.* **2022**, *12* (14), 8300–8309.
